# A comprehensive analysis of prognostic signatures reveals the high predictive capacity of the Proliferation, Immune response and RNA splicing modules in breast cancer

**DOI:** 10.1186/bcr2192

**Published:** 2008-11-13

**Authors:** Fabien Reyal, Martin H van Vliet, Nicola J Armstrong, Hugo M Horlings, Karin E de Visser, Marlen Kok, Andrew E Teschendorff, Stella Mook, Laura van 't Veer, Carlos Caldas, Remy J Salmon, Marc J van de Vijver, Lodewyk FA Wessels

**Affiliations:** 1 Department of Pathology, The Netherlands Cancer Institute, Plesmanlaan 121, 1066 CX Amsterdam, The Netherlands; 2 Department of Surgery, Institut Curie, 6 rue d'Ulm, 75005 Paris, France; 3 UMR 144, CNRS-Institut Curie, Molecular Oncology Team, 12 rue Lhomond, 75005 Paris, France; 4 Bioinformatics and Statistics Group, The Netherlands Cancer Institute, Plesmanlaan 121, 1066 CX Amsterdam, The Netherlands; 5 Faculty of EEMCS, Delft University of Technology, Mekelweg 4, 2628 CD Delft, The Netherlands; 6 Department of Molecular Biology, The Netherlands Cancer Institute, Plesmanlaan 121, 1066 CX Amsterdam, The Netherlands; 7 Cancer Research UK, Cambridge Research Institute and Department of Oncology, Li Ka Shing Centre, University of Cambridge, Cambridge CB2 ORE, UK; 8 Department of Pathology, Academic Medical Center, Meibergdreef 9, 1100 DD Amsterdam The Netherlands

## Abstract

**Introduction:**

Several gene expression signatures have been proposed and demonstrated to be predictive of outcome in breast cancer. In the present article we address the following issues: Do these signatures perform similarly? Are there (common) molecular processes reported by these signatures? Can better prognostic predictors be constructed based on these identified molecular processes?

**Methods:**

We performed a comprehensive analysis of the performance of nine gene expression signatures on seven different breast cancer datasets. To better characterize the functional processes associated with these signatures, we enlarged each signature by including all probes with a significant correlation to at least one of the genes in the original signature. The enrichment of functional groups was assessed using four ontology databases.

**Results:**

The classification performance of the nine gene expression signatures is very similar in terms of assigning a sample to either a poor outcome group or a good outcome group. Nevertheless the concordance in classification at the sample level is low, with only 50% of the breast cancer samples classified in the same outcome group by all classifiers. The predictive accuracy decreases with the number of poor outcome assignments given to a sample. The best classification performance was obtained for the group of patients with only good outcome assignments. Enrichment analysis of the enlarged signatures revealed 11 functional modules with prognostic ability. The combination of the RNA-splicing and immune modules resulted in a classifier with high prognostic performance on an independent validation set.

**Conclusions:**

The study revealed that the nine signatures perform similarly but exhibit a large degree of discordance in prognostic group assignment. Functional analyses indicate that proliferation is a common cellular process, but that other functional categories are also enriched and show independent prognostic ability. We provide new evidence of the potentially promising prognostic impact of immunity and RNA-splicing processes in breast cancer.

## Introduction

Breast cancer is composed of distinct diseases with different outcomes. Clinical and pathological factors are currently employed to determine the prognosis of patients. The Saint-Gallen guidelines [1], National Institute of Health guidelines [2] and Nottingham Prognostic Index guidelines [3] as well as the AdjuvantOnline! decision-making tools [4] use a combination of these prognostic factors to adapt adjuvant treatment based on the prognosis prediction . Owing to insufficiently accurate prognosis predictions, however, a substantial proportion of breast cancer patients with breast cancer of inherently good outcome receive adjuvant systemic therapy without gaining any benefit [5].

High-throughput technologies such as gene expression microarrays can offer new opportunities to improve the ability to determine individual prognosis. After confirmation of their performance in validation studies, classifiers such as the 70-gene signature developed at the Netherlands Cancer Institute [6,7] and the 76-gene signature developed at the Erasmus Medical Center [8,9] may become of use in clinical practice. Similarly, Paik and colleagues built a 16-gene classifier for paraffin-embedded tissues (OncotypeDX^©^; Genomic Health, Redwood, California, USA) using quantitative RT-PCR [10]. The ongoing MINDACT trial [11] and TAILORx trial [12] aim to confirm the performance of the 70-gene and the OncotypeDX signatures on large populations of 6,000 patients and 10,000 patients, respectively. In the meantime, it has become commonplace for research groups to define new classifiers with a potentially higher level of performance than existing classifiers [13-21].

Publications have raised several concerns about microarray studies that would potentially impair the use of gene expression classifiers in clinical routine [22-24]. We performed a comprehensive analysis of gene-expression-based classifiers on a collection of 1,127 breast cancer samples all hybridized on the Affymetrix^© ^platform. To the best of our knowledge, this represents the largest, single platform dataset employed to evaluate prognostic classifiers to date.

Nine gene expression signatures derived using diverse methodological approaches and focusing on various aspects thought to be associated with breast cancer outcome were selected: the 76-gene signature employed to compute the Relapse score [8]; the 52-gene and 17-gene signatures employed to compute the Molecular Prognostic Index (T52 and T17) [20]; the Intrinsic/UNC gene set used to define the molecular subtypes [15]; the 70-gene and 25-gene Chromosomal Instability signatures (CIN70 and CIN25) [13]; the Core Serum Response signature [14]; the Invasiveness Gene Signature [16]; and the 97-gene signature used to derive the Gene expression Grade Index [25]. (Note that there are nine signatures in total, but T17 and CIN25 are smaller versions of T52 and CIN70, respectively. T17 and CIN25 were therefore not included in the enlargement and enrichment analyses.) Two well-known signatures, the 21-gene Genomic Health signature [10] and the 70-gene signature from the Netherlands Cancer Institute [6,7], were not included. The Genomic Health signature was not included since it is not a micro-array-based gene expression signature but is designed for RT-PCR assays. The 70-gene signature was not included since the 295 sample dataset on which this signature was developed was employed as a completely independent validation set. Throughout the text we use signature to refer to both the gene set as well as the classifier, which, based on this gene set, assigns a sample to an outcome group.

While some signatures were designed for specific subgroups of patients, we applied the signatures to the complete, unstratified set of breast cancer samples. We followed this approach for several reasons. First, a large dataset maximizes power to detect common prognostic factors, while stratification into subtypes results in smaller groups and, therefore, in reduced power. Second, there is mounting evidence that classifiers designed within a specific subgroup also perform well in other subgroups. For example, the 70-gene classifier developed by van 't Veer and colleagues was developed for lymph-node-negative patients [6], but performs very well on lymph-node-positive patients as demonstrated by van de Vijver and colleagues [7]. In fact, patients with up to three positive lymph nodes are now included in the MINDACT trial [26]. Similarly, the 21-gene OncotypeDX signature, which was developed for node-negative disease, has also been shown to be a good predictor of disease outcome in node-positive patients [27]. These facts indicate that breast cancer subgroups as defined by classical markers are currently being re-evaluated, and that the signatures have some common core of biological/molecular processes involved in the development of breast cancer that is critical for predicting good outcome or poor outcome. Finally, an analysis performed on the complete dataset provides a benchmark against which subtype specific signature performance can be gauged and also reveals whether predictors designed on the whole cohort have any predictive value in subgroups.

We addressed the following issues: Do these nine separate signatures perform similarly? Are there (common) molecular processes reported by these signatures? Can better prognostic predictors be constructed based on (a combination of) these identified molecular processes?

## Materials and methods

**Figure 1:**
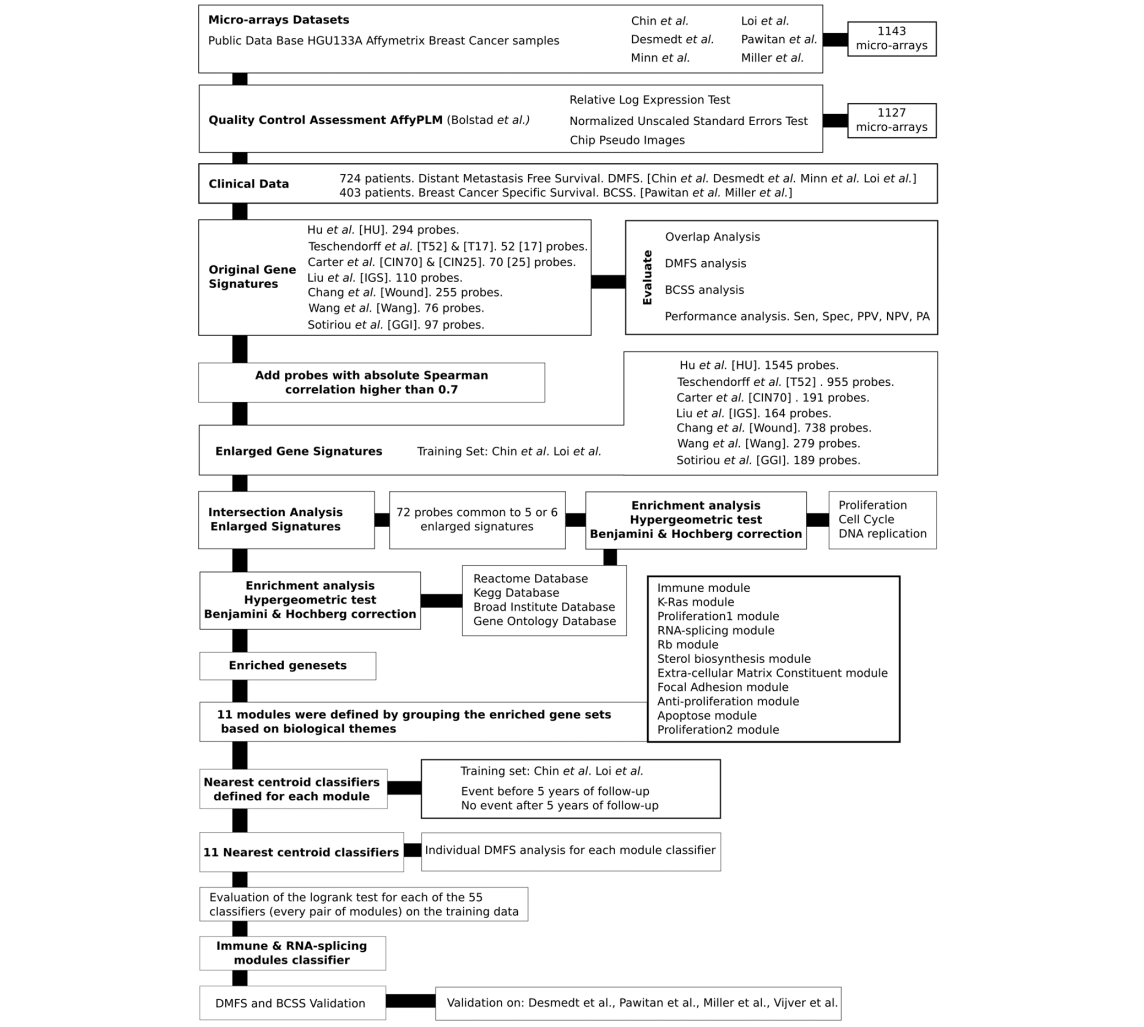
**Detailed overview of the datasets, gene sets and analysis steps employed**. PPV, positive predictive value; NPV, negative predictive value; PA, predictive accuracy.

Figure 1 depicts a graphical overview of the analysis procedure followed. A detailed Materials and methods section is available in Additional data file 1.

### Data preprocessing

Six breast cancer datasets [9,17-19,28,29] – all arrayed on the same platform (HGU-133A Affymetrix^© ^Santa Clara, California, USA) to avoid cross-platform discrepancies – for which the raw data (.CEL files) were publicly available were downloaded from the Gene Expression Omnibus and ArrayExpress repository websites [30,31] . This resulted in a total of 1143 microarrays. Of these, 1,127 were deemed to be of sufficient quality and were kept in the current study. Sixteen micro-arrays were rejected due to major artifacts in the original .CEL files.

To ensure comparability between the different datasets, they were all subjected to the same preprocessing procedure. Microarray quality-control assessment was carried out using the AffyPLM R-package [32]. Selected arrays were normalized using the RMA expression measure algorithm [33]. For 724 samples, distant metastasis-free survival (DMFS) data were available; while for 403 samples, breast cancer-specific survival (BCSS) data were available. For some samples both DMFS and BCSS information is available. For the performance analyses, where the results per endpoint are reported separately, the overlapping samples were analyzed twice (once for each endpoint).

For the enlargement of the signatures and the construction of the functional classifiers, only the Chin and Loi datasets were used – resulting in a total of 450 samples. We will refer to this combined dataset as the Chin–Loi training set. Clinical and histopathological features for all samples are summarized in Table 1.

**Table 1:** Clinical and histopathological features of 1,127 patients presenting breast carcinoma and included in the survival analyses

	Distant metastasis-free survival	Breast cancer-specific survival
Patients (n)	724	403
Tumor size		
Number (%) of T1 (≤ 20 mm)	292 (43%)	271 (69%)
Mean (standard deviation) size (mm)	25.3 (13)	22.2 (10.4)
Histological grading: Elston Ellis		
Grade I	104 (15.4%)	88 (23%)
Grade II	253 (37.5%)	176 (45%)
Grade III	195 (28.9%)	111 (28%)
Not available	121 (17.9%)	13 (3%)
Genomic grade index		
High grade	331 (49%)	179 (46%)
Estrogen receptor status: immunohistochemistry		
Positive	488 (72.5%)	197 (51%)
Not available	6 (0.8%)	160 (40%)
Estrogen receptor status: gene expression		
Positive	496 (74%)	297 (76%)
Her2 status: gene expression		
Positive	86 (13%)	50 (13%)
Lymph node metastasis		
Positive	197 (28%)	76 (20%)
Not available	8 (1%)	165 (41%)
Distant metastasis or death from breast cancer		
Positive	177 (26%)	83 (21%)
Within 5 years	131 (19%)	59 (15%)
Mean (standard deviation) follow-up (months)	85.6 (53.4)	89.6 (39.8)
Datasets used (Ref)	[9,17,19,29]	[18,28]

Sixty-six patients out of 1,127 samples were excluded from the survival analysis due to missing information (time or event censoring).

### Signature validation

To the complete dataset of 1,127 samples, we applied the 76-gene signature [8], the Intrinsic/UNC signature [15], the Chromosomal Instability signatures (CIN70 and CIN25) [13], the Core Serum Response signature [14], the Invasiveness Gene Signature [16], the Molecular Prognosis Index signatures (T52 and T17) [20], and the Gene expression Grade Index signature [25]. Survival analyses were performed using the Kaplan–Meier estimate of the survival function. Comparison between survival curves was performed using the log-rank test. Hazard ratios were estimated using the Cox proportional hazard model. *P *values were considered significant when <0.05. Only variables with a significant *P *value in univariate analyses were included in a multivariate model. Time-censoring analyses were performed using right censoring of the events from 1 to 12 years. The performance analysis (sensitivity, specificity, positive predictive value, negative predictive value, and predictive accuracy) of the signatures was carried out using the ROCR package [32] . For these analyses, the outcome was dichotomized into a poor outcome group (samples with an event before 5 years of follow-up) and a good outcome group (samples with no event and a follow-up of at least 5 years). The analyses were performed using R software [32] and Matlab software [34].

### Enlarged signatures

We created enlarged signatures by including all probes with an absolute Spearman rank order correlation >0.7 with at least one of the genes in the original signatures. This calculation was performed on the Chin–Loi training set.

### Enrichment analyses of signatures

For all signatures, we evaluated whether specific gene sets (that is, functional groups), are overrepresented. We gathered a collection of 5,480 gene sets from four databases: Gene Ontology [35], Kyoto Encyclopedia of Genes and Genomes [36], Reactome [37] and the Molecular Signatures Database [38] (see Materials and methods in Additional data file 1) . The hypergeometric test was employed to test the significance of the overlap between each signature and gene set. Multiple testing adjustment was performed by applying the Benjamini–Hochberg correction (per signature) [39].

### Discovery and validation of module classifiers

The gene sets that were enriched in at least one signature were grouped into modules based on common functional annotation. The only exception was the group of proliferation-related gene sets. This group was split into two groups, the first containing gene sets common to either five or six enlarged signatures while the second contained the remaining proliferation gene sets. This resulted in 11 functional modules. For each of these modules we constructed a nearest mean classifier using the genes common between the enlarged signatures and the group of genes in the module. The classifier was trained using the Chin–Loi training set. For each module, the poor outcome centroid was derived from samples with a metastatic event before 5 years of follow-up; the good outcome centroid was derived from samples with no metastatic events and a follow-up longer than 5 years. Based on the genes associated with a given module, the Spearman rank correlation between each sample and the centroid values of the poor outcome and the good outcome centroids were determined. Each sample in the training set was then assigned to the centroid with which it showed the highest correlation (the nearest centroid).

We then created classifiers by combining the output of every pair of module classifiers. For each pair of modules, this results in a three-class classifier: samples with two poor (good) group labels are assigned to the poor (good) group, while samples with discordant labels are assignment to the intermediate group. The performance of these classifiers was evaluated, based on the log-rank test, on the time-to-event data on the Chin–Loi training set. The best module-pair classifier was selected to enter the validation stage.

We validated the best module-pair classifier on independent data. Validation was performed for DMFS on the datasets of Desmedt and colleagues [9] and Minn and colleagues [19], while for BCSS the datasets of Pawitan and colleagues [28] and Miller and colleagues [18] were used . In addition, we validated the best module-pair classifier on the dataset of van de Vijver and colleagues [7]. The gain in predictive accuracy of the classifier, as compared with common clinical staging systems, was investigated on the dataset of van de Vijver and colleagues using the method of Schemper and Henderson [40], implemented in the R package software [41] . Dunkler and colleagues have also recently used this approach in the context of gene signatures [42]. Briefly, predictive inaccuracy is calculated as the average of the absolute difference between observed outcomes and the model predictions. The explained variation is also computed and represents a measure equivalent to *R*^2 ^in linear regression. Standard errors were obtained by bootstrapping 200 resamples.

## Results

### Prognostic performance

**Figure 2:**
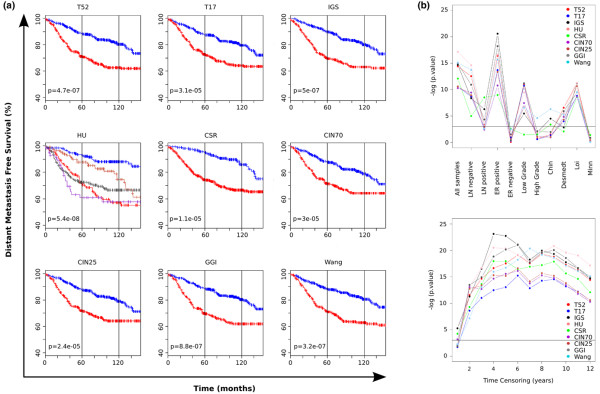
**Kaplan–Meier analysis of distant metastasis-free survival**. **(a) **Analysis performed for 724 patients. In all cases except the Intrinsic/UNC subtyping gene set (HU) the blue (red) curve represents the good (poor) outcome group. Each panel depicts the result obtained after applying one of the classifiers (indicated in the heading) as described in Materials and methods. Represented signatures: T17 and T52, Molecular Prognostic Index signature [20]; IGS, Invasiveness Gene signature [16]; HU, Intrinsic/UNC gene set [15] – resulting in the Luminal A (blue), Normal-like (coral), Luminal B (red), Basal (grey) and Her2 (purple) subtypes – CSR, Core Serum Response signature [14]; CIN70 and CIN25, Chromosomal Instability signature [13]; GGI, Gene expression Grade Index [25]; and Wang, 76-gene signature [8]. **(b) **Top panel: performance of the signatures on subgroups of the patient population. Vertical axis, -log(*P *value) of the log-rank test from the Kaplan–Meier analysis for a particular subgroup with distant metastasis-free survival (DMFS) as the endpoint. Analyzed subgroups: lymph node (LN)-negative, LN-positive, estrogen receptor (ER)-positive, ER-negative, low grade (Elston Ellis I), high grade (Elston Ellis III), individual Chin, Desmedt, Loi and Minn datasets. Horizontal line, *P *= 0.05. Bottom panel: time-censoring performance analysis of the signatures. Horizontal axis, time at which right censoring was applied to all samples; vertical axis, -log(*P *value) of the log-rank test from the Kaplan–Meier analysis for a given time-censoring and a particular signature with DMFS as the endpoint. Horizontal line, *P *= 0.05.

Cox proportional hazard analyses using clinical and histological features with DMFS and BCSS as endpoints are summarized in Additional data file 1. Nine gene signatures were applied to the complete dataset of 1,127 breast cancer samples (Figure 2). Each of the signatures was used to define a poor outcome group and a good outcome group. For both clinical endpoints (DMFS, BCSS), the Kaplan–Meier curves obtained for each of the nine signatures were very similar – the exception being the Intrinsic/UNC gene set, which, per definition, defines five molecular subtypes (Figure 2a; see also Additional data file 2, Figure S1a). The log-rank test for differences in survival between the different groups as classified by the individual signatures was significant (*P *< 0.05) in all cases.

In addition, multivariate Cox proportional hazard models were fitted with lymph node (LN) status, size, estrogen receptor (ER) status (DMFS only) and each signature separately (see Additional data file 1, Table S2). All nine signatures remained as significant variables in the multivariate models for DMFS. Tumor size was the most significant, in terms of *P *value, in all models except for those including the Intrinsic/UNC gene set signature and the 76-gene signature. When considering BCSS, three signatures remained significant: Intrinsic/UNC gene set (*P *= 0.01), Invasiveness Gene Signature (*P *= 0.02) and Core Serum Response signature (*P *= 0.039). The clinical variables LN and size were significant in all models considered.

The data were then divided into subsets according to LN status, ER status, grade and origin, and the signatures were compared in terms of their association with survival in these clinical subgroups (Figure 2b (top panel); see also Additional data file 2, Figure S1b (top panel)). With DMFS as the endpoint, no signature was significantly associated with survival in the ER-negative subgroup. In the LN-positive subgroup, some signatures were significantly associated with survival – while in the high-grade subgroup, only a single signature was significantly associated with DMFS. The sample sizes in these subgroups are relatively small (<200 patients); however, the individual hazard ratios (HRs) for the signatures within these subgroups are of similar magnitude. For BCSS, only the ER-positive subgroup showed association and was the largest subgroup available for this endpoint (n = 297). Other clinical subgroups in this analysis have small sample sizes (between 80 and 160 patients). We also applied the signatures to the individual datasets, and found that no signature showed association with survival in the Minn dataset (Figure 2b (top panel); see also Additional data file 2, Figure S1b (top panel)). For this dataset, only 80 samples had survival information; however, all signatures had HRs close to 1.

The sensitivity of the signatures to variations in the time after which all events are censored was also investigated. Over a censoring range of 1 year to 12 years, each of the nine signatures showed a very similar pattern (Figure 2b (bottom panel); see also Additional data file 2, Figure S1b (bottom panel)) specific to the clinical endpoint of interest. With DMFS as the endpoint, the performance measure reached a maximal value at approximately 5 years. This might be explained by the fact that some of the signatures were generated using a 5-year cutoff point to define the good and poor outcome groups. Two validation series of the 76-gene signature [9] and of the 70-gene signature [43] also showed strong time-dependent performance variability. The hazard ratio adjusted for clinical parameters, however, was still significant for the censoring time ranging from 1 year to 10 years.

### Classification concordance

**Figure 3:**
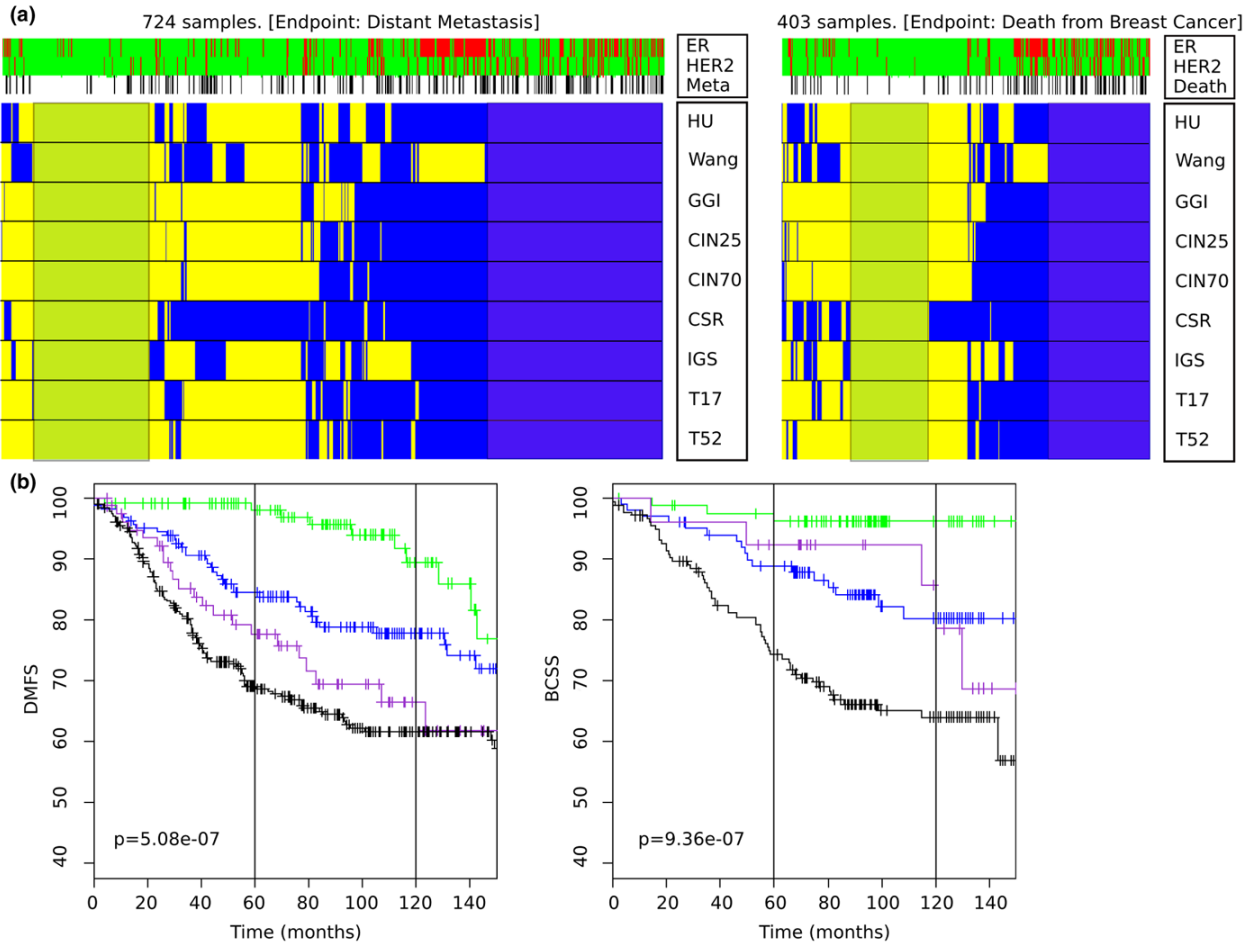
**Classification and stratification of poor outcome for distant metastasis-free survival and breast cancer-specific survival**. **(a) **Classification results of the nine gene signatures on the collection of 1,127 samples with distant metastasis-free survival (DMFS) (left panel) and breast cancer-specific survival (BCSS) (right panel) as the endpoints. In both panels, breast cancer samples are depicted in columns, and signatures in rows. Each cell colored according to the outcome of the signature in the corresponding row for the patient in the associated column: blue, poor outcome; yellow, good outcome. Top three lanes represent clinical parameters. HER2, HER2 status with red representing amplified cases and green nonamplified cases (as determined from gene expression); ER, estrogen receptor status with red representing ER-negative cases and green ER-positive cases as determined by gene expression; Meta or Death, DMFS or BCSS with events indicated in black. Represented signatures: HU, Intrinsic/UNC gene set [15]; IGS, Invasiveness Gene signature [16]; CIN70 and CIN25, Chromosomal Instability signatures [13]; GGI, Gene expression Grade Index [25]; T17 and T52, Molecular Prognostic Index signature [20]; CSR, Core Serum Response signature [14]; and Wang, the 76-gene signature [8]. For the subtyping based on the Intrinsic/UNC gene set, assignment to the Luminal B, Basal and HER2 subtypes was scored as poor outcome while assignment to the Luminal A and Normal types was scored as good outcome. Shaded regions highlight those patients classified as good outcome or poor outcome by all signatures. **(b) **Kaplan–Meier curves for the population of 1,127 samples stratified according to the number of times a sample is assigned to the poor outcome category with DMFS (left panel) and BCSS (right panel) as the endpoints. Graphs depict the curves for four subgroups: all good group (no poor outcome assignments; green), mainly good group (one to three poor outcome assignments; blue), mainly poor group (four to six poor outcome assignments; purple), and poor group (seven to nine poor outcome assignments; black).

For each signature, every sample was labeled either good outcome or poor outcome. Figure 3a provides an overview of the assignments of the samples to either outcome group by all signatures for DMFS (left panel) and for BCSS (right panel).

We investigated the concordance of the classification labels for the nine signatures. Considering DMFS as the endpoint, only 322 out of 724 samples (44%) had perfect concordance: 127 samples (17.5%) were consistently classified as good outcome, while 195 samples (26.9%) were classified as poor outcome by all signatures. These samples are indicated by the shaded regions in Figure 3a. A total of 114 samples (15.7%) proved difficult to classify since more than one-third of the signatures assigned discordant labels to these samples. When BCSS was taken as the endpoint (n = 403), similar results were found: 198 (49%) had perfect concordance, with 86 samples (21.3%) always classified as good outcome and 112 samples (27.8%) always classified as poor outcome by the signatures. A number of samples (46 samples, 11.4%) were again difficult to classify, with more than one-third of the signatures assigning discordant labels to them. In order to investigate the effect of sample heterogeneity on the discordance, we repeated this analysis for subgroups based on ER status, LN status, and their combinations. The results are depicted in Additional data file 3. This analysis revealed that the concordance/discordance percentages within these subgroups are always similar. That is, the discordance ranged from 52% to 62% for DMFS and from 44% to 60% for BCSS, leading to the conclusion that the concordance/discordance is not due to these clinical parameters.

**Figure 4:**
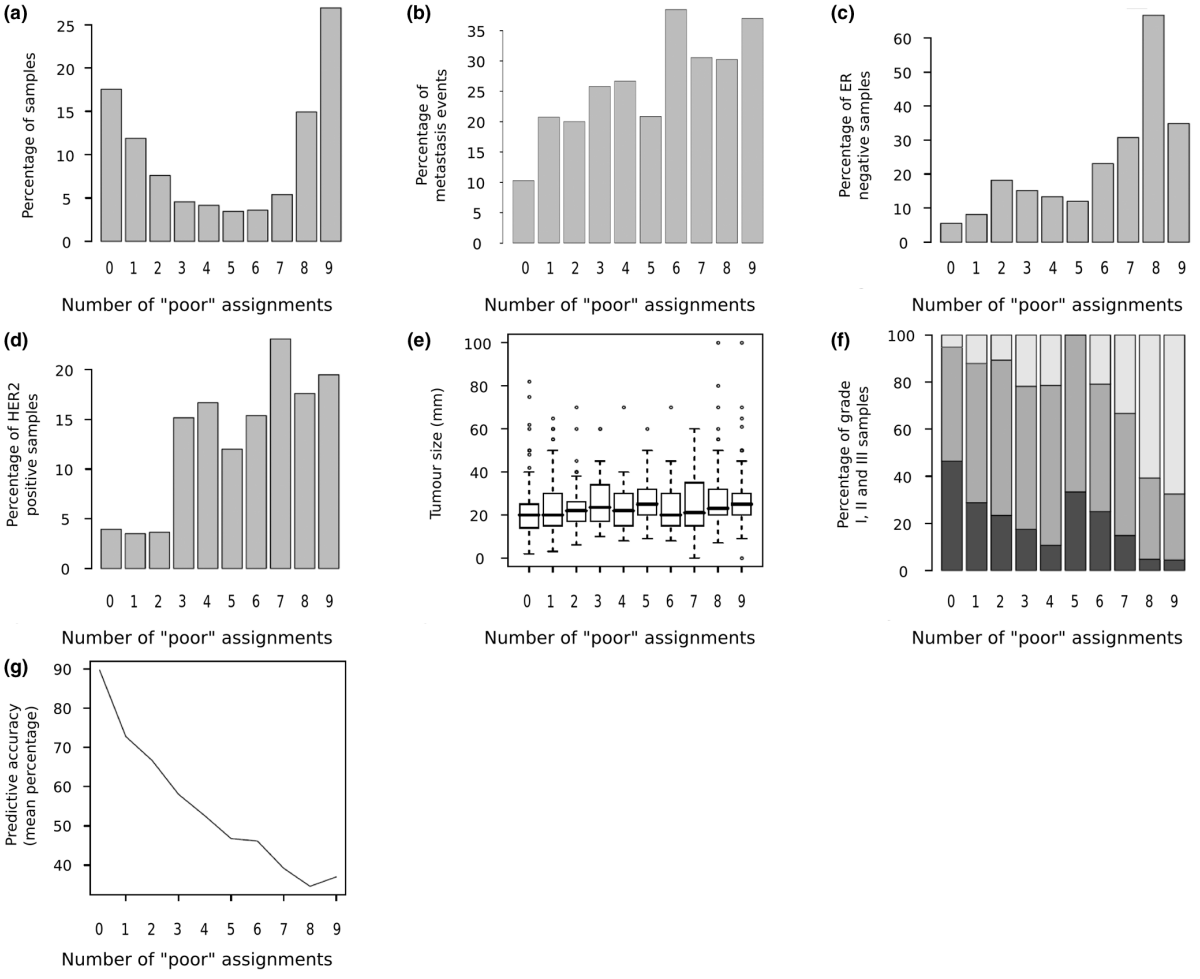
**Overlap and performance analysis of 724 samples with distant metastasis-free survival as the endpoint**. **(a) **Distribution of the samples as a function of the number of times a sample was classified as of poor prognosis by the gene signatures. **(b) **Distribution of metastasis events as a function of the number of times a sample was classified as of poor prognosis by the gene expression signatures. **(c) **Distribution of estrogen receptor (ER)-negative samples as a function of the number of times a sample was classified as of poor prognosis by the gene signatures. **(d) **Distribution of HER2 amplified samples as a function of the number of times a sample was classified as of poor prognosis by the gene signatures. **(e) **Tumor size (mean, 5th, 25th, 75th and 95th percentiles) as a function of the number of times a sample was classified as of poor prognosis by the gene signatures. **(f) **Distribution of grade I, grade II and grade III samples (Elston Ellis) as a function of the number of times a sample was classified as of poor prognosis by the gene signatures. Dark shading, grade I; grey shading, grade II; pale shading, grade III. **(g) **Average predictive accuracy (percentage of samples well classified) as a function of the number of times a sample was classified as of poor prognosis by the gene signatures.

Several clinical parameters are associated with the number of poor outcome assignments. The results for DMFS are depicted in Figure 4, while the results for BCSS are presented in Additional data file 4. In both cases, the proportion of events was significantly correlated with the number of times each sample was assigned to the poor outcome group (DMFS, chi-square test, *P *= 1.9 × 10^-4^; BCSS, chi-square test, *P *= 6.1 × 10^-10^). Similarly, the proportion of patients with ER-negative status (DMFS, *P *= 2.2 × 10^-16^; BCSS, *P *= 2 × 10^-14^), with HER2-positive status (DMFS, *P *= 8.5 × 10^-5^; BCSS, *P *= 0.0001), in the high-grade subgroup (DMFS, *P *= 2.2 × 10^-16^; BCSS, *P *= 2.2 × 10^-16^) and with a mean tumor size (DMFS, *P *= 0.0004; BCSS, *P *= 2.4 × 10^-5^) were all correlated to the number of times a sample was classified as poor outcome by the nine signatures.

To further investigate the impact of the number of poor outcome classifications, the samples were divided into four categories: all good (no poor outcome), mainly good (one to three poor outcomes), mainly poor (four to six poor outcomes), and poor (seven or more poor outcomes). Using this grouping, Kaplan–Meier analysis was performed using DMFS as the endpoint (Figure 3b (left panel), log-rank, *P *= 5.1 × 10^-7^). A univariate Cox proportional hazard model showed significantly increased HRs when comparing the all good group with the other groups (versus mainly good group, HR = 2.15 (1.11 to 4.14); versus mainly poor group, HR = 3.49 (1.73 to 7.01); versus poor group, HR = 4.12 (2.27 to 7.49)). Using BCSS as the endpoint, the log-rank test was again significant (*P *= 9.4 × 10^-7^; Figure 3b (right panel)) and the HRs increased significantly when comparing the all good group with the other groups (data not shown).

The sensitivity, specificity, positive predictive value, negative predictive value and predictive accuracy were very similar for all signatures – except for the Core Serum Response signature, which showed higher sensitivity and lower specificity (see Additional data file 1). The predictive accuracy (fraction of correctly assigned samples) of each signature was analyzed as a function of the number of times a sample was assigned to the poor outcome group. For both endpoints, the predictive accuracy is very high for the samples always classified as good outcome (DMFS, 87%; BCSS, 96%) and decreases dramatically with the number of poor outcome assignments. The predictive accuracy for the samples with nine poor outcome labels was only 45% for DMFS and 36% for BCSS (see Figure 4 and Additional data file 4).

### Enlarged gene signature analysis

The intersection of seven gene signatures showed that no probe set was present in more than four signatures and that most of the probe sets were found in only one signature. In order to better reveal the common processes associated with the signatures, each of the signatures was enlarged by augmenting them with highly correlated probes (absolute Spearman correlation >0.7). These enlarged signatures contain, on average, three times as many probes as the original signatures. We identified a group of 72 probe sets present in at least five enlarged gene signatures. This overlap is highly significant, since observing an overlap of 72 or more probes between the two enlarged signatures with the smallest number of genes (164 genes, enlarged Invasiveness Gene Signature; and 189 genes, enlarged Gene expression Grade Index signature) is already highly significant (*P *<2.2 × 10^-16^, hypergeometric test). The chance of observing an overlap of 72 or more probes among all seven enlarged signatures will be even smaller, and thus even more significant. An enrichment analysis of these 72 probe sets showed an overrepresentation of genes related to DNA replication, cell cycle, and proliferation.

### Constructing and validating module classifiers

**Figure 5:**
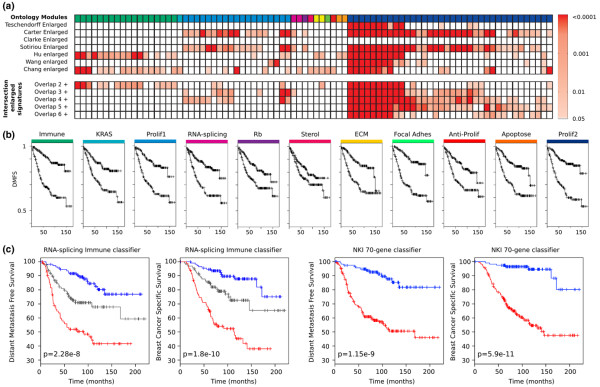
**Enrichment analysis for the seven enlarged signatures and Kaplan–Meier analysis of distant metastasis-free survival**. **(a) **Enrichment analysis for the seven enlarged signatures on a collection of 1,889 gene sets from the Reactome, Kyoto Encyclopedia of Genes and Genomes (KEGG), Molecular Signatures Database, and Gene Ontology databases. Significance of the *P *values was computed with the hypergeometric test. All *P *values were adjusted for multiple testing using the Benjamini–Hochberg method. Each cell in the matrix represents the adjusted *P *value for a given enlarged signature and a gene set. The ontology modules to which a particular gene set belongs are indicated at the top. See (b) for the link between the color coding and the module identity. **(b) **Kaplan–Meier analysis of distant metastasis-free survival (DMFS) on the Chin–Loi training set for each of the 11 ontology modules. The Kaplan–Meier analysis is based on the output of a nearest mean classifier trained on the genes in each of the ontology modules. **(c) **Kaplan–Meier analysis of DMFS on van Vijver and colleagues' breast cancer series as stratified by the Immune and RNA splicing module classifier, for both endpoints. Blue curve, low-risk group where both classifiers assign a patient to the good outcome group; grey curve, intermediate-risk category where the classifiers are discordant; red curve, high-risk category where both classifiers assign a patient to the poor outcome group. For comparison, the same dataset as stratified by the Netherlands Cancer Institute (NKI) 70-gene classifier for both endpoints is also presented.

Functionally related gene sets were merged to define functional modules. This resulted in the following 11 modules: Immune, KRAS, Proliferation1 (defined by genes common to two to four enlarged signatures), Proliferation2 (defined by genes common to five or more enlarged signatures), RNA splicing, Rb pathway, Sterol biosynthesis, Extracellular matrix constituent (ECM), Focal adhesion, Negative regulation of proliferation, and Apoptosis (Figure 5a). For each of these modules we determined a nearest centroid classifier on the Chin–Loi training set. On this training set, with DMFS as the endpoint, all modules except Sterol biosynthesis had a significant association with outcome (log-rank test *P *value ranging from 4.4 × 10^-3 ^to 6.8 × 10^-9^; Figure 5b).

We then set out to construct a classifier that combines the binary class assignments of the module classifiers into a single, three-valued (low-risk, intermediate-risk and high-risk) classification output. We restricted the search to pairs of modules, since the size of the discordant groups (where at least one module classifier output differs from the others) grows as the number of modules combined increases, leading to difficulty in interpretability. The separate classifiers were combined in a pairwise fashion (55 possible combinations) and the resulting classifiers were evaluated on the Chin–Loi training set. The classifier with the most significant log-rank test (lowest *P *value) on the training data was selected. This classifier was based on the Immune and RNA splicing modules (see Additional data files 5 and 6). Samples that are assigned to the good outcome (poor outcome) category by both the Immune and the RNA splicing module classifiers are classified as low (high) risk, while the discordant cases are assigned to the intermediate risk category. On independent data, this classifier was a significant prognosis predictor (data not shown) of DMFS (dataset of Desmedt and colleagues [9]) and BCSS (datasets of Pawitan and colleagues [28] and Miller and colleagues [18]).

The Immune and RNA splicing classifier was then validated using the dataset of van de Vijver and colleagues [7]. For both DMFS and BCSS, we found a highly significant association between the classifier and outcome (log-rank test: DMFS, *P *= 2.28 × 10^-8^; BCSS, *P *= 1.8 × 10^-10^) (Figure 5c). For DMFS, the HR for the intermediate-risk group versus the low-risk group is 2.25 (1.3 to 3.9), while the HR for the high-risk group versus the low-risk group is 4.35 (2.57 to 7.36). Similarly, for BCSS the HR of the intermediate-risk group versus the low-risk group was 2.31 (1.2 to 4.5) and the HR for the high-risk group versus the low-risk group is 6.1 (3.3 to 11.4). The known clinical predictors Elston Ellis grading, age at diagnosis, size (mm) and ER status, as well as the Immune and RNA splicing classifier, were then included in a multivariate analysis. For both endpoints, the combined classifier was the most significant variable in the model (see Additional data file 1).

The gain in predictive accuracy from adding the RNA splicing/Immune signature to each of the three common clinical staging systems is presented in Table 2. The largest decrease in predictive inaccuracy is seen when the RNA splicing/Immune signature is added to the St Gallen index, as this has the worst individual performance for both DMFS and BCSS (0.31 and 0.287, respectively). In this case, the predictive accuracy is reduced from 0.287 to 0.254 for BCSS (DMFS, from 0.310 to 0.282). In terms of explained variation, the St Gallen index is again the worst, explaining only 1% to 2% of the variation in this dataset. With 6% to 8% of the explained variation being attributable to the Nottingham Prognostic Index dataset, it is the best of the clinical staging systems. In contrast, the RNA splicing/Immune signature explains a larger amount of the variation (10% to 13%) on its own, and including it with one of the three staging systems improves on this only marginally. For this dataset, there is a gain in the predictive accuracy when adding the RNA splicing/Immune classifier to existing predictors. Additional data file 7 shows the prognostic capacity of the signature conditional on the levels of the different clinical staging systems.

**Table 2:** Explained variation and predictive inaccuracy for distant metastasis-free survival and breast cancer-specific survival in the 295 Netherlands Cancer Institute dataset

Model	Distant metastasis-free survival		Breast cancer-specific survival	
		
	Predictive inaccuracy	Explained variation (%)	Predictive inaccuracy	Explained variation (%)
No predictors	0.314 ± 0.02			0.292 ± 0.02
Adjuvant online	0.303 ± 0.02	3.5 ± 1.8	0.274 ± 0.02	6.1 ± 2.0
Nottingham Prognostic Index	0.294 ± 0.02	6.2 ± 2.4	0.270 ± 0.02	7.8 ± 2.6
St Gallen	0.310 ± 0.02	1.2 ± 0.9	0.287 ± 0.02	1.9 ± 1.0
RNA splicing/Immune module classifier	0.282 ± 0.02	9.9 ± 3.0	0.254 ± 0.02	13.0 ± 3.8
Adjuvant online and module classifier	0.281 ± 0.02	10.3 ± 2.9	0.250 ± 0.02	14.5 ± 3.8
Nottingham Prognostic Index and module classifier	0.277 ± 0.02	11.6 ± 3.2	0.248 ± 0.02	15.2 ± 3.8
St Gallen and module classifier	0.282 ± 0.02	9.9 ± 3.0	0.254 ± 0.02	13.1 ± 3.7
Gain by adding module classifier to Adjuvant online	0.022	6.8	0.024	8.4
Gain by adding module classifier to Nottingham Prognostic Index	0.017	5.4	0.022	7.4
Gain by adding module classifier to St Gallen	0.028	8.7	0.033	11.2

Data presented as the mean ± standard error.

## Discussion

The analyses performed here demonstrate that each of the nine gene expression signatures have similar classification performance based on the sensitivity, specificity, negative predictive value, positive predictive value and predictive accuracy. All gene expression signatures added independent information to a multivariate model including standard pathological and clinical criteria. Although the gene expression classifiers were mostly defined to determine the risk of distant metastasis events and not the risk of death from breast cancer, we found similar results for each gene classifier when using either DMFS or BCCS as the endpoint.

The Gene expression Grade Index [25] and the molecular prognostic index signatures (T17 and T52) [20] were developed for ER-positive breast cancer, while the 76-gene expression classifier signature [8] was defined for LN-negative tumors. Despite these prerequisites, we applied the nine gene expression classifiers to the same dataset without any consideration for the heterogeneity of these samples as our first goal was to compare these different classifiers when applied to the same dataset. More importantly, the generalization of subgroup-specific classifiers (for example, LN0 classifiers) across a complete cohort of breast cancer samples (both LN0 and LN1) hints at the existence of common biological processes determining the outcome. We were interested in revealing these processes. Furthermore, when evaluating the signatures in specific subgroups of patients, we showed similar behavior for each of these nine gene signatures. No signature showed strong association with survival when applied to LN-positive, ER-negative or high-grade subgroups. These results are potentially explained by the fact that these factors identify a set of intrinsically poor outcome cases; that is, they contain no good outcome cases. This emphasizes the fact that gene expression classifiers should, in our opinion, not be regarded as a tool to replace standard pathological and clinical criteria, but should instead be integrated with clinical parameters. Gene expression classifiers can be employed to improve stratification in subgroups of breast cancer patients with good prognosis, where the groups are defined based on standard pathological and clinical criteria.

Fan and colleagues [24] showed similar performance and significant concordance between the 70-gene signature [6,7], the Core Serum Response signature [14], the Genomic Health signature [10] and the Intrinsic/UNC gene set [15] when applied to the dataset of van de Vijver and colleagues [7]. In contrast, we show here that agreement between gene expression signatures is low, with >50% of the samples having at least one discordant class assignment. We showed that the predictive accuracy dramatically decreases with the number of poor prognostic assignments a sample receives. The best classification performance was obtained for the group of patients with only good outcome assignments. These results immediately reveal the dilemma faced by a patient diagnosed with breast cancer, and determines consultation of a collection of signatures to predict disease outcome. The result obtained is uncertain in almost 50% of the cases. As our results were less optimistic than those of Fan and colleagues [24], we repeated our analyses as described above but this time used the dataset of van de Vijver and colleagues [7] and the following signatures: the 70-gene signature [6,7] (employing Fan and colleagues' labeling [24]), the Core Serum Response signature [14], the Genomic Health signature [10], the Intrinsic/UNC gene set [15], and the Gene expression Grade Index [25]. The results recapitulated our earlier results. In particular, only 42% of the samples received a concordant class assignment, while the ER status, HER2 status, pathological grading and tumor size were all correlated with the number of times a sample was classified as poor outcome by the signatures. As was demonstrated earlier, the predictive accuracy decreased with the number of poor outcome assignments. Larger datasets (such as those acquired in the TAILORx and MINDACT trials [11,12] are required to shed more light on the cases where the signatures give discordant class assignments.

To gain more insight into the small degree of overlap between the genes comprising the different classifiers, we generated an enlarged signature for each signature. The intersection of the enlarged signatures identified a core of 72 genes significantly enriched in DNA replication, cell cycle and mitosis ontology annotations, which we consider the common background of these gene signatures. Proliferation genes are a major component of many prognostic signatures in breast carcinoma and other tumor types [44]. Among the 72 genes we found *AURKA*, *BIRC5*, *CCNB1*, *MKI67 *and *MYBL2*, which define the complete set of proliferation genes from the Genomic Health signature [10]. The proliferation modules also contain genes frequently described as markers of proliferation in different types of cancer [45]: *PLK1*, *BUB1*, *CCNA2*, *CCNB1*, *CCNB2*, *CCNE2*, *FOXM1*, and *TOP2A*. These genes are derived from the functional intersection of the enlarged gene expression signatures, indicating that proliferation is a major driver of the prognosis gene signatures.

The enrichment analysis of the enlarged signatures revealed 11 gene ontology modules. Identification of distinct biological processes correlated with survival or other clinicopathological features is a major step towards improving our understanding of tumor development and to providing accurate information to develop new targeted therapies. Yu and colleagues generated 500 gene signatures of ER-positive and ER-negative tumors [46], and found the following pathways to be overrepresented in the signatures: apoptosis, proliferation, focal adhesion, RNA splicing and immunity. They emphasized that similar pathways are common to different gene signatures, whereas the individual genes defining these pathways can still have varying degrees of association with outcome.

We showed that the combination of the Immune and RNA splicing modules define a classifier that is highly accurate in predicting both DMFS and BCSS. In addition, the classifier showed an improvement in predictive accuracy when combined with commonly used clinical staging systems. This indicates that not only proliferation but also other functional processes have prognostic power. Teschendorff and colleagues recently showed that the overexpression of a seven-gene immunity module is associated with good outcome in 186 ER-negative breast cancers [21]. No significant correlation between lymphocyte infiltration and this immunity module was found. Two of these seven genes (*XCL2 *and *HLA-F*) are also in our classifier. Recent clinical and experimental studies have revealed that not only cancer cell intrinsic processes, but also cancer cell extrinsic processes – including angiogenesis, remodeling of the extracellular matrix, and inflammation – are critical in determining malignant outcome. The role of the immune system during cancer progression has recently gained much attention [47]. The reciprocal interaction between the immune system and cancer can be regarded as a double-edged sword: whereas certain interactions inhibit or prevent cancer growth, other interactions actually contribute to tumor progression. For example, *in situ *analysis of tumor-infiltrating lymphocytes in human colorectal cancer samples revealed that the influx of T lymphocytes is associated with improved survival, and the immunological data were found to be a better predictor of patient survival than the histopathological methods currently used to stage colorectal cancer [48].

The RNA splicing process is a key molecular event in the generation of protein biodiversity. Alteration of the normal process results in the production of altered mRNA or in an off-balance production of tissue-specific mRNA isoforms [49-51]. The main consequences of this abnormal RNA splicing process are a reduction of the normal protein level or the production of abnormal proteins. Aberrant mRNA splicing variants are found in many cancers and can interfere with major biological events such as apoptosis, cell-cycle control, adhesion, differentiation or angiogenesis. Mutations in splicing cis-acting sequences have been associated with the *BRCA1 *gene in breast cancer [52] and the *KIT *oncogene in gastrointestinal stromal tumor [53]. The RNA splicing module we identified contains several genes that are individually strongly associated with survival. More specifically, *SFRS10 *is significantly overexpressed in breast cancer and might be responsible for splicing of CD44 isoforms associated with tumor progression and metastasis [54]. *SRPK1 *is upregulated in breast cancer and its expression level is proportional to the tumor grade. Inhibition of *SRPK1 *results in reduced phosphorylation of *MAPK3*, *MAPK1 *and *AKT *[55]. Targeted *SRPK1 *treatment seems to be a promising way to increase apoptosis, to decrease proliferation and to enhance the sensitivity to chemotherapeutics drugs [56]. *LSM1 *is located at 8p11-12 loci and is amplified in almost 20% of breast cancer cases [57]. Streicher and colleagues showed that overexpression of *hLsm1 *transforms mammary epithelial cells, and inhibition of its expression in breast cancer cells reduces anchorage-independent proliferation [58]. Yang and colleagues similarly showed the same ability of *LSM1 *to transform human mammary epithelial cells *in vitro *[57].

## Conclusion

In the present article we set out to address three questions regarding the signatures considered in the study. First, we can conclude that the nine gene expression signatures had similar performance. This was observed for both the accuracy with which samples were assigned to the dichotomous poor/good outcome groups as well as the level of association with survival found. Nevertheless, the concordance of outcome assignment between gene expression signatures is low, with 50% of the samples receiving at least one outcome assignment that is discordant with the assignments of the other signatures. This relatively high level of gene expression classification instability is associated with a dramatic decrease in predictive accuracy with an increase in the number of poor outcome assignments. The heterogeneity in the outcome assignments of the different classifiers can most probably be attributed to the different approaches that were followed to construct the classifiers, the heterogeneity in the sample populations employed to construct the classifiers, and sample size issues [59].

In the present study we showed that the common background of the nine gene signatures investigated is a 72-gene cluster. Eleven gene ontology modules were overrepresented in the enlarged signatures. These modules revealed a wide array of functional groups that were overrepresented in gene sets highly correlated with the probes contained in the original signatures. Finally, we demonstrated that the combination of the Immune and RNA splicing modules defined an efficient classifier for breast cancer. This result shows that pathway-level analysis of microarrays is able to provide a functionally coherent and highly efficient prognosis classifier, which will most probably be more stable than the classifiers from which it originates.

## Abbreviations

BCSS: breast cancer-specific survival; DMFS: distant metastasis-free survival; ER: estrogen receptor; HR: hazard ratio; LN: lymph node; PCR: polymerase chain reaction; RT: reverse transcriptase.

## Competing interests

LvV is employed by Agendia BV, has an ownership interest in MammaPrint, and is the named inventor on a patent to use microarray technology to ascertain breast cancer prognosis and holds equity interests in Agendia BV. The other authors declare that they have no competing interests.

## Authors' contributions

FR and MHvV contributed equally to this work, carrying out the study design, data mining, data analysis and manuscript writing. NJA carried out the study design, data mining, data analysis and manuscript writing. HMH carried out the study design and data mining. KEdV carried out the data analysis and manuscript writing. MK carried out the study design and manuscript writing. AET carried out the Molecular Prognosis Index calculation. SM carried out the study design and manuscript advising. LvV carried out the manuscript advising. CC carried out the Molecular Prognosis Index calculation. RJS carried out the fund raising and manuscript advising. MJvdV carried out the study design, data analysis and manuscript writing. LFAW carried out the study design, data mining, data analysis and manuscript writing.

## Supplementary Material

Additional file 1 A Word file containing the supplementary Materials and methods section and four supplementary tables. Table S1 lists data for multivariate and univariate Cox regression analyses with DMFS and BCSS as the endpoints. Table S2 lists data for multivariate Cox regression analysis with selected clinical parameters – ER status based on immunohistochemistry (DMFS only), LN status (positive versus negative), histological grading (Elston Ellis I, II and III) – and the output of the nine gene expression classifiers as input and either DMFS and BCSS as clinical endpoints. Table S3 lists a performance analysis of the signatures on the complete set of 1,127 patients with dichotomous outcome labels of poor outcome and good outcome derived from DMFS and BCSS. Table S4 lists data for multivariate Cox regression analysis with selected clinical parameters – ER status based on immunohistochemistry, LN status (positive versus negative), histological grading (Elston Ellis I, II and III) – tumor size and the output of the Immune and RNA splicing modules gene signature (IR) or the 70-gene signature (NKI) as the input and DMFS and BCSS analysis as the clinical endpoint. Click here for file

Additional file 2 An Adobe file containing a figure of the RNA splicing, Immune and 72 Proliferation gene annotations [Probe_ID, EntrezID, OMIM, Ensembl, UnigeneID, Representative Public ID, RefSeq Transcript ID, Gene Symbol, k-means metastasis, k-means no metastasis]. Click here for file

Additional file 3 An Adobe file containing a figure showing the nine signature Kaplan–Meier curves with BCSS as endpoints (S1a), showing the performance of the signatures on subgroups of the patient population (S1b (top panel)), and showing) the time-censoring performance analysis of the signatures (1b (bottom panel). Click here for file

Additional file 4 An image file containing a figure showing heatmaps of the concordance of the nine classifiers across clinical subgroups among the 1,127 human breast tumor samples. Click here for file

Additional file 5 An image file containing a figure showing the overlap and performance analysis of 403 samples with BCSS as the endpoint. Click here for file

Additional file 6 An Excel file containing a figure showing the log-rank test *P *value on the Chin–Loi training set for each pairwise combination of the module classifiers. Click here for file

Additional file 7 An Adobe file containing a figure showing the Kaplan–Meier plots on the dataset of van de Vijver and colleagues for the subgroups defined by the Nottingham Prognostic Index, St Gallen, and AdjuvantOnline! clinical staging systems – plots for the Immune/RNA splicing module classifier within each of the clinical subgroups for each staging system. Click here for file
