# Supplementary material for: A comprehensive analysis of prognostic signatures reveals the high predictive capacity of the Proliferation, Immune response and RNA splicing modules in breast cancer
Source: Breast Cancer Res. 2008 Nov 13;10(6):R93. doi: 10.1186/bcr2192 (PMC2656909; doi:10.1186/bcr2192)
Supplement: Additional file 1 — A Word file containing the supplementary Materials and methods section and four supplementary tables. Table S1 lists data for multivariate and univariate Cox regression analyses with DMFS and BCSS as the endpoints. Table S2 lists data for multivariate Cox regression analysis with selected clinical parameters – ER status based on immunohistochemistry (DMFS only), LN status (positive versus negative), histological grading (Elston Ellis I, II and III) – and the output of the nine gene expression classifiers as input and either DMFS and BCSS as clinical endpoints. Table S3 lists a performance analysis of the signatures on the complete set of 1,127 patients with dichotomous outcome labels of poor outcome and good outcome derived from DMFS and BCSS. Table S4 lists data for multivariate Cox regression analysis with selected clinical parameters – ER status based on immunohistochemistry, LN status (positive versus negative), histological grading (Elston Ellis I, II and III) – tumor size and the output of the Immune and RNA splicing modules gene signature (IR) or the 70-gene signature (NKI) as the input and DMFS and BCSS analysis as the clinical endpoint. [file bcr2192-S1.doc]

**Supplemental Material for Reyal et al.**

**Material and Methods**

**Data preprocessing**

We collected all publicly available datasets containing raw gene expression micro-array data of breast cancer samples. In order to avoid cross-platform discrepancies, the study was limited to Human Genome HGU-133A Affymetrix© arrays. The raw data were downloaded from NCBI’s Gene Expression Omnibus with the following identifiers; GSE6532 (Loi *et al.(17)*), GSE3494 (Miller *et al.(18)*), GSE1456 (Pawitan *et al.(28)*), GSE7390 (Desmedt *et al.(9)*) and GSE5327 (Minn *et al.(19)*). The Chin *et al.(29)* data set was downloaded from ArrayExpress (identifier E-TABM-158).

To ensure comparability between the different datasets, they were all subjected to the same pre-processing procedure. Micro-array quality-control assessment was carried out using the R AffyPLM package available from the Bioconductor web site. We applied the Relative Log Expression (RLE) and Normalized Unscaled Standard Errors (NUSE) tests. Chip pseudo-images were produced to assess artefacts on arrays that didn't pass the preceding quality control tests.

Selected arrays were normalized according to a three step procedure using the RMA expression measure algorithm : RMA background correction convolution, median centering of each gene across arrays separately for each data set and quantile normalization of all arrays.

**Determination of ER/Her2 status**

The oestrogen receptor (ER) gene expression status was determined using the 205225_at probe set. For 947 out of 1127 samples, ER status determined by immuno-histochemistry (IHC) was available. A gene-expression cut-off of 1.834 resulted in a 90% sensitivity in determining the ER status (IHC). All samples with a gene expression value higher than 1.834 were classified as ER positive. Similarly, the Her2 gene expression status was determined using the 216836_s_at probe set. A density plot of the 1127 gene expression values showed a bimodal distribution. The lowest value of the density plot between the two modes determined the cut-off between the Her2 positive and Her2 negative status. All samples with a gene expression value higher than 1.62 were classified as Her2 positive.

**Signature validation**

On the complete dataset of 1127 samples, we applied the 76-gene signature [Wang](8); Intrinsic/UNC gene set to define the ‘Molecular subtypes’ [Hu](15); Chromosomal Instability Signatures [CIN70, CIN25](13); Core Serum Response signature [CSR](14); Invasiveness Gene Signature [IGS](16); Molecular Prognosis Index signature [T52, T17](21) and Genne expression Grade Index [GGI](17)*.*

The classifier defined by Wang *et al.*(8) was applied as described in their publication. Since it was defined on the HG-U133A Chip, there was no need for probe mapping. ER status is included in this signature, hence we used the ER status as determined by gene expression. The constants A and B (from the relapse score in Wang *et al.*) were recalculated to center the relapse score to zero for both ER positive and ER negative patients. Patients with a score greater than or equal to zero were classified as ‘poor prognosis’, patients with a score lower than zero were classified as ‘good prognosis’.

Hu *et al.*(15) defined and validated centroids of 306 genes to discriminate between 5 previously identified breast cancer subtypes (Luminal A and B, Basal, Her2, Normal). We matched the probe list UniGene ID (Build#204) to the HG-U133A Affymetrix© platform. A total of 549 HGU-133A probe sets were identified corresponding to these 306 genes. As several probe sets corresponded to the same intrinsic gene, a single probe set was kept for the analysis. Probe sets with an ‘‘_at’’ extension were preferentially kept because they tend to be more specific according to Affymetrix© probe set design algorithms. Probe sets with an ‘‘_s_at’’ extension were the second best choice followed by all other extensions. When several probe sets with the same extension were available for one gene, the one with the highest inter-quartile range of expression values across all arrays was kept. This resulted in a list of 294 unique HGU-133A probe sets. Each sample was assigned to the nearest subtype/centroid as determined by the highest Spearman rank order correlation between the gene expression values of the 294 probes sets and the five subtype centroids.

The two Chromosomal INstability signatures (CIN)(13) are composed of 70 and 25 genes, respectively. The probe list UniGene ID (Build#204) was matched to the HG-U133A Affymetrix© platform. We found 103 HGU-133A corresponding probe sets. As several probe sets corresponded to the same gene, a single probe set was kept for the analysis, as described above, resulting in lists of 70 and 25 unique HGU-133A probe sets. The CIN70 (CIN25) score was determined for each sample by the sum of the 70 (25) gene expression probe set values. A ‘poor prognosis’ CIN70 (CIN25) signature was defined by a score higher than the mean of all CIN70 (CIN25) scores obtained from all the samples.

The CSR signature(15) is a 512 gene signature. After matching the probe list UniGene ID (Build#204) to the HG-U133A Affymetrix© platform, 258 HGU-133A corresponding probe sets were identified. The core serum response score was calculated by the Pearson correlation between the 258 gene expression values for each sample and the core serum response centroid. An ‘Activated’ signature was assigned when the score was higher than -0.15 as defined.

The Invasiveness Gene Signature (IGS)(16) is a 186 gene signature composed of 110 HGU-133A probe sets and 76 HGU-133B probe sets. The IGS score was determined by calculating the Pearson correlation between the 110 HGU-133A probe set expression values of each sample with the 110 reference expression levels of the same genes defining the IGS signature. An IGS score higher than 0 was considered as ‘poor prognosis’.

The two Molecular Prognosis Indexes(21) and the Genomic Grade Index (GGI)(17) were applied as described,.

**Statistical analysis of survival and the performance of the 9 gene signatures**

Survival analyses were performed using the Kaplan Meier estimate of the survival function. Comparison between survival curves was performed using the logrank test. The endpoints of these analyses were distant metastasis free survival (DMFS) (all metastasis events) and breast cancer specific survival (BCSS) (death from breast cancer). Estimation of hazard ratios was carried out using the Cox proportional hazard model. P-values were considered significant when below 0.05. Only variables with a significant p-value in univariate analyses were included in a multivariate model. Time censoring analyses were performed using a right censoring of the events from 1 to 12 years. The analysis was performed using R software. The performance analysis was carried out using the ROCR package. Sensitivity, specificity, positive predictive value and negative predictive value were estimated by the area under curves obtained by ROC analysis. To estimate the performance of the Hu *et al.*(15) Molecular Subtypes we considered the Luminal A and Normal like as the ‘good prognosis’ class and the Luminal B, Basal and Her2 as the ‘poor prognosis’ class.

**Enlarged signatures**

Since the genes in a signature are not unique, they can be replaced by other predictive genes without significant changes in performance of the classifier (23). This has been put forward as one of the reasons for the limited overlap between gene signatures. Therefore, we sought to generate more complete signatures of genes that are related to outcome by extending the signatures with highly correlated genes. More specifically, we created enlarged signatures by including all probes with an absolute Spearman rank order correlation higher than 0.7 relative to at least one of the genes in the original signature. This calculation was performed on the merged Chin *et al.(29)* and Loi *et al.*(17) datasets. For this analysis, the two Chromosomal Instability Signatures (CIN70 and CIN25)(13) were considered as one 70-gene signature and the two Molecular Prognosis Index (T52 and T17)(21) were considered as one 52-gene signature. The intersection between the seven original signatures and the intersection between the seven enlarged signatures was assessed in terms of overlapping Affymetrix© probe set IDs.

**Functional enrichment of the signatures**

For all signatures, we evaluated whether specific gene sets (i.e. functional groups), are overrepresented. We gathered a collection of 5480 gene sets from four databases: Gene Ontology (Function and Process trees, 4745 gene sets), KEGG (187 gene sets), Reactome (26 gene sets) and the MSDB (C2, 522 gene sets).

For the entire HGU-133A array, we mapped the probes to Entrez IDs , resulting in a total of 12962 unique Entrez IDs present on the array. Only probes with an ‘_at’, ‘_s_at’,and ‘_x_at’ extension were used. Successively, all signatures were mapped to Entrez IDs. For the enrichment analysis we only used gene sets with at least five annotated Entrez IDs that are also present on the array, resulting in 1889 gene sets. We used the hypergeometric test to test the significance of the overlap between each signature and gene set. Multiple testing correction was taken into account by applying the Benjamini and Hochberg correction (per signature)(36).

**Discovery and validation of the Immune and RNA-splicing classifier**

The intersection analysis of the seven enlarged signatures (probe set ID) resulted in a list of 72 probe sets. The gene sets that were enriched were then grouped based on common functional annotation into modules. This resulted in 11 functional modules (composed of gene sets enriched in at least one enlarged signature and one pathway). The identified modules are: Immune; KRAS; Proliferation1 (defined by genes common to 2 to 4 enlarged signatures); Proliferation2 (defined by genes common to 5 or 6 enlarged signatures); RNA splicing; Rb Pathway; Sterol biosynthesis; Extra-cellular matrix constituent; Focal adhesion; Negative regulation of proliferation and Apoptosis. For each of these 11 modules we constructed a nearest mean classifier based on the merged *Chin et al. (29)* and *Loi et al.(17)* datasets. The genes comprising each of these 11 modules were obtained by taking the intersection between the union of genes belonging to all gene sets within a module, and the set of genes, which comprise at least one signature. This leads to a set of Entrez IDs for each module. We selected a single Affymetrix© HGU-133A probe for each Entrez ID in the following manner: First probes were favoured based on the Affymetrix algorithm probe extension (favoring ‘_at’ over ‘_x_at’ over ‘_s_at’). When multiple probes with the same extension map to an Entrez ID we selected the one with the highest variance (in the *Chin et al.(29)* and *Loi et al.(17)* datasets). For each module, the ‘poor prognosis’ centroid was derived from the samples with a metastasic event before 60 months of follow-up. The ‘good prognosis’ centroid was derived from the samples with no metastasic events and a follow-up longer than 60 months. Each sample in the independent validation set was assigned to the nearest centroid as determined by the highest Spearman rank order correlation score between the gene expression value of the corresponding probe sets of each sample and the centroid values of the ‘poor prognosis’ and ‘good prognosis’ centroid for each of the 11 modules. A final classifier was constructed by combining the Immune module and the RNA-splicing module, resulting in a three-class classifier, stratifying the samples in high, medium and low risk categories. We validated this classifier on independent data consisting of the *Desmedt et al.(9)* and *Minn et al.(19)* datasets (with DMFS as endpoint) and the *Pawitan et al.(28)* and *Miller et al.(18)* datasets (with BCSS as endpoint). In addition, we validated the same centroid classifiers on the series of 295 breast cancer samples from the Netherlands Cancer Institute(7). To facilitate this, the Rosetta© reporter IDs were mapped to the corresponding Entrez IDs. When multiple reporters mapped to the same Entrez ID, we selected the probe with the highest variance.

**Supplemental table 1**

Multi- and univariate Cox regression analyses with distant metastasis free survival (DMFS) and breast cancer specific survival (BCSS) as endpoints

| Distant Metastasis Free Survival Analysis | | | | Breast Cancer Specific Survival Analysis | | | |
| --- | --- | --- | --- | --- | --- | --- | --- |
| **Variables** | **N** | **Hazard Ratio** | **p** | **Variables** | **N** | **Hazard Ratio** | **p** |
| **Univariate Analysis Clinical and Histological Features** | | | | | | | |
| *Lymph Node Status* | 665 | 1.84 (1.35-2.52) | 0.000109 | *Lymph Node Status* | 223 | 4.26 (2.46-7.39) | 2.4e-07 |
| *Size (mm)* | 670 | 1.03 (1.02-1.04) | 1e-08 | *Size (mm)* | 232 | 1.05 (1.03-1.07) | 9.34e-06 |
| *Elston Ellis II vs I* | 552 | 1.97 (1.13-3.45) | 0.017 | *Elston Ellis II vs I* | 375 | 3.22 (1.44-7.18) | 0.0043 |
| *Elston Ellis III vs I* | 552 | 2.32 (1.32-4.1) | 0.0036 | *Elston Ellis III vs I* | 375 | 5.18 (2.29-11.75) | 0.00008 |
| *Estrogen Receptor* | 667 | 0.58 (0.43-0.8) | 0.000615 | *Estrogen Receptor* | 228 | 1.1 (0.47-2.57) | 0.825 |
| **Multivariate Analysis Clinical and Histological Features** | | | | | | | |
| *Lymph Node Status* | 538 | 1.67 (1.12-2.41) | 0.01 | *Lymph Node Status* | 221 | 2.8 (1.52-5.14) | 0.00086 |
| *Size (mm)* | 538 | 1.03 (1.01-1.04) | 0.0001 | *Size (mm)* | 221 | 1.03 (1.007-1.06) | 0.011 |
| *Elston Ellis II vs I* | 538 | 1.64 (0.93-2.89) | 0.087 | *Elston Ellis II vs I* | 221 | 1.61 (0.69-3.76) | 0.27 |
| *Elston Ellis III vs I* | 538 | 1.41 (0.75-2.63) | 0.29 | *Elston Ellis III vs I* | 221 | 2.15 (0.83-5.54) | 0.11 |
| *Estrogen Receptor* | 538 | 0.62 (0.41-0.93) | 0.021 |  |  |  |  |

**Supplemental table 2**

Multivariate Cox regression analysis with selected clinical parameters - Estrogen Receptor (ER) status based on immuno-histochemistry (DMFS only), lymph node (LN) status (positive vs. negative), histological grading (Elston Ellis I, II and III) - and the output of the nine gene expression classifiers as input and either distant metastasis free survival (DMFS) and breast cancer specific survival (BCSS) as clinical end points.

**Legend:**

# T17 & T52: Molecular Prognostic Index signature (Teschendorff *et al.*)

# IGS: invasiveness signature (Liu *et al.*);

# Lum A: Luminal A subtype according to Hu *et al.*

# Lum B: Luminal B subtype according to Hu *et al.*

# CSR: Core Serum Response signature (Chang *et al.*).

# CIN70 & CIN25: Chromosomal instability signature (Carter *et al.*).

# GGI: Gene expression Grade Index (Sotiriou *et al.*, GGI)

# Wang: The 76-gene signature (Wang *et al.*).

| Distant Metastasis Free Survival Analysis | | | | Breast Cancer Specific Survival Analysis | | | |
| --- | --- | --- | --- | --- | --- | --- | --- |
| **Variables** | **N** | **Hazard Ratio** | **p** | **Variables** | **N** | **Hazard Ratio** | **P** |
| **Multivariate analysis. Estrogen Receptor status (IHC), Lymph Node status, Tumor size (mm) plus one gene signature** | | | | | | | |
| *Estrogen receptor* | 656 | 0.79 (0.57-1.1) | 0.17 |  |  |  |  |
| *Lymph Node status* | 656 | 1.51 (1.08-2.14) | 0.016 | *Lymph Node status* | 223 | 3.15 (1.75-5.65) | 0.0068 |
| *Tumor Size* | 656 | 1.019 (1.009-1.03) | 0.00021 | *Tumor Size* | 223 | 1.03 (1.009-1.06) | 0.00011 |
| *Teschendorff 52* | 656 | 1.906 (1.366-2.66) | 0.00015 | *Teschendorff 52* | 223 | 1.56 (0.87-2.81) | 0.13 |
| *Estrogen receptor* | 656 | 0.7 (0.53-1.02) | 0.068 |  |  |  |  |
| *Lymph Node status* | 656 | 1.51 (1.074-2.12) | 0.018 | *Lymph Node status* | 223 | 3.19 (1.78-5.71) | 0.000092 |
| *Tumor Size* | 656 | 1.02 (1.01-1.03) | 0.00013 | *Tumor Size* | 223 | 1.03 (1.009-1.06) | 0.006 |
| *Teschendorff 17* | 656 | 1.65 (1.19-2.3) | 0.0027 | *Teschendorff 17* | 223 | 1.7 (0.94-3.06) | 0.076 |
| *Estrogen receptor* | 656 | 0.77 (0.55-1.07) | 0.12 |  |  |  |  |
| *Lymph Node status* | 656 | 1.5 (1.067-2.12) | 0.02 | *Lymph Node status* | 223 | 2.86 (1.59-5.16) | 0.00047 |
| *Tumor Size* | 656 | 1.018 (1.008-1.03) | 0.0005 | *Tumor Size* | 223 | 1.03 (1.01-1.06) | 0.0074 |
| *IGS* | 656 | 1.79 (1.28-2.51) | 0.00062 | *IGS* | 223 | 2.57 (1.38-4.77) | 0.0028 |
| *Estrogen receptor* | 656 | 0.67 (0.45-1.01) | 0.058 |  |  |  |  |
| *Lymph Node status* | 656 | 1.43 (1.02-2.02) | 0.038 | *Lymph Node status* | 223 | 2.77 (1.52-5.03) | 0.00079 |
| *Tumor Size* | 656 | 1.019 (1.009-1.03) | 0.00032 | *Tumor Size* | 223 | 1.04 (1.017-1.07) | 0.00063 |
| *LumB vs. LumA* | 656 | 3.469 (2.086-5.77) | 0.0000017 | *LumB vs. LumA* | 223 | 1.5 (0.65-3.45) | 0.34 |
| *Normal-like vs. LumA* | 656 | 2.215 (1.246-3.94) | 0.0067 | *Normal-like vs. LumA* | 223 | 1.01 (0.4-2.52) | 0.98 |
| *Basal-like vs. LumA* | 656 | 2.237 (1.25-4.01) | 0.0067 | *Basal-like vs. LumA* | 223 | 1.09 (0.47-2.48) | 0.84 |
| *Her2-like vs. LumA* | 656 | 3.526 (1.881-6.61) | 8.4e-05 | *Her2-like vs. LumA* | 223 | 3.02 (1.25-7.29) | 0.014 |
| *Estrogen receptor* | 656 | 0.70 (0.51-0.96) | 0.03 |  |  |  |  |
| *Lymph Node status* | 656 | 1.49 (1.06-2.107) | 0.022 | *Lymph Node status* | 223 | 3.23 (1.82-5.74) | 0.000063 |
| *Tumor Size* | 656 | 1.019 (1.009-1.03) | 0.00026 | *Tumor Size* | 223 | 1.03 (1.01-1.06) | 0.0035 |
| *CSR* | 656 | 2.232 (1.43-3.486) | 0.00041 | *CSR* | 223 | 2.46 (1.05-5.77) | 0.039 |
| *Estrogen receptor* | 656 | 0.759 (0.54-1.06) | 0.1 |  |  |  |  |
| *Lymph Node status* | 656 | 1.52 (1.082-2.14) | 0.016 | *Lymph Node status* | 223 | 3.22 (1.79-5.75) | 0.000083 |
| *Tumor Size* | 656 | 1.02 (1.01-1.03) | 0.00013 | *Tumor Size* | 223 | 1.03 (1.008-1.06) | 0.0085 |
| *CIN70* | 656 | 1.628 (1.171-2.26) | 0.0038 | *CIN70* | 223 | 1.58 (0.88-2.83) | 0.12 |
| *Estrogen receptor* | 656 | 0.76 (0.54-1.06) | 0.11 |  |  |  |  |
| *Lymph Node status* | 656 | 1.52 (1.082-2.14) | 0.016 | *Lymph Node status* | 223 | 3.2 (1.78-5.72) | 0.000091 |
| *Tumor Size* | 656 | 1.02 (1.01-1.03) | 0.00013 | *Tumor Size* | 223 | 1.03 (1.009-1.06) | 0.0068 |
| *CIN25* | 656 | 1.659 (1.93-2.31) | 0.0026 | *CIN25* | 223 | 1.51 (0.84-2.69) | 0.16 |
| *Estrogen receptor* | 656 | 0.796 (0.57-1.11) | 0.19 |  |  |  |  |
| *Lymph Node status* | 656 | 1.498 (1.06-2.11) | 0.02 | *Lymph Node status* | 223 | 3.15 (1.76-5.65) | 0.00012 |
| *Tumor Size* | 656 | 1.019 (1.01-1.03) | 0.00021 | *Tumor Size* | 223 | 1.03 (1.01-1.06) | 0.0075 |
| *GGI* | 656 | 1.813 (1.3-2.53) | 0.00044 | *GGI* | 223 | 1.64 (0.93-2.9) | 0.087 |
| *Estrogen receptor* | 656 | 0.623 (0.456-0.85) | 0.0029 |  |  |  |  |
| *Lymph Node status* | 656 | 1.496 (1.061-2.11) | 0.021 | *Lymph Node status* | 223 | 3.09 (1.73-5.52) | 0.00014 |
| *Tumor Size* | 656 | 1.019 (1.008-1.03) | 0.00041 | *Tumor Size* | 223 | 1.04 (1.014-1.06) | 0.0013 |
| *Wang* | 656 | 2.025 (1.476-2.78) | 0.000013 | *Wang* | 223 | 1.5 (0.85-2.62) | 0.16 |

**Supplemental table 3**

Performance analysis of the signatures on the complete set of 1127 patients with dichotomous outcome labels being ‘poor’ outcome and ‘good’ outcome derived from distant metastasis free survival (DMFS) and breast cancer specific survival (BCSS). The Data are censored after five years of follow-up.

**Legend:**

# T17 & T52: Molecular Prognostic Index signature (Teschendorff *et al.*).

# IGS: Invasiveness Gene Signature (Liu *et al.*).

# Lum A: Luminal A subtype according to Hu *et al.*

# Lum B: Luminal B subtype according to Hu *et al.*

# CSR: Core Serum Response signature (Chang *et al.*).

# CIN70 & CIN25: chromosomal instability signature (Carter *et al.*).

# GGI: Gene expression Grade Index (Sotiriou *et al.*, GGI).

# Wang: The 76-gene signature 'Relapse score' (Wang *et al.*).

# Sen: Sensitivity.

# Spec: Specificity*.*

# PPV: Positive predictive value.

# NPV: Negative predictive value.

PA: Predictive accuracy

|  | **DMFS** | | | | | **BCSS** | | | | |
| --- | --- | --- | --- | --- | --- | --- | --- | --- | --- | --- |
|  | **Sen** | **Spec** | **PPV** | **NPV** | **PA** | **Sen** | **Spec** | **PPV** | **NPV** | **PA** |
| **T52** | 0.66 | 0.57 | 0.42 | 0.78 | 0.6 | 0.73 | 0.58 | 0.34 | 0.88 | 0.62 |
| **T17** | 0.66 | 0.53 | 0.40 | 0.77 | 0.57 | 0.75 | 0.57 | 0.34 | 0.88 | 0.61 |
| **IGS** | 0.66 | 0.56 | 0.41 | 0.78 | 0.59 | 0.75 | 0.60 | 0.36 | 0.89 | 0.64 |
| **Hu** | 0.73 | 0.53 | 0.42 | 0.80 | 0.59 | 0.73 | 0.59 | 0.35 | 0.88 | 0.62 |
| **CSR** | 0.87 | 0.29 | 0.37 | 0.83 | 0.48 | 0.90 | 0.34 | 0.29 | 0.92 | 0.47 |
| **CIN70** | 0.63 | 0.56 | 0.40 | 0.76 | 0.58 | 0.71 | 0.58 | 0.34 | 0.87 | 0.61 |
| **CIN25** | 0.64 | 0.55 | 0.40 | 0.77 | 0.58 | 0.71 | 0.58 | 0.34 | 0.87 | 0.61 |
| **GGI** | 0.63 | 0.59 | 0.42 | 0.77 | 0.6 | 0.70 | 0.62 | 0.36 | 0.87 | 0.64 |
| **Wang** | 0.67 | 0.55 | 0.41 | 0.78 | 0.59 | 0.65 | 0.57 | 0.31 | 0.84 | 0.59 |

**Supplemental Table 4**

*Multivariate Cox regression analysis with selected clinical parameters - Estrogen Receptor status based on immuno-histochemistry, lymph node status (positive vs. negative), histological grading (Elston Ellis I, II and III) - tumor size and the output of the Immune and RNA-splicing modules gene signature (IR) or the 70-gene signature (NKI) as input and distant metastasis free survival and breast cancer specific survival analysis as clinical end point.*

| Distant Metastasis Free Survival Analysis | | | | Breast Cancer Specific Survival Analysis | | | |
| --- | --- | --- | --- | --- | --- | --- | --- |
| **Variables** | **N** | **Hazard Ratio** | **p** | **Variables** | **N** | **Hazard Ratio** | **p** |
| **Multivariate Clinical, Histological and Combined Gene Signature Analysis (Immune and RNA-splicing modules)** | | | | | | | |
| *Size in mm* | 295 | 1.02 (1.00-1.05) | 0.045 | *Size in mm* | 295 | 1.02 (0.99-1.04) | 0.17 |
| *Elston Ellis Grade II vs. I* | 295 | 1.87 (0.93-3.77) | 0.078 | *Elston Ellis Grade II vs. I* | 295 | 3.57 (1.21-10.49) | 0.02 |
| *Elston Ellis Grade III vs. I* | 295 | 2.20 (1.07-4.52) | 0.033 | *Elston Ellis Grade III vs. I* | 295 | 4.54 (1.53-13.53) | 0.0065 |
| *Estrogen receptor* | 295 | 1.01 (0.63-1.62) | 0.96 | *Estrogen receptor* | 295 | 0.59 (0.36-0.97) | 0.037 |
| *Lymph Node* | 295 | 1.22 (0.81-1.82) | 0.34 | *Lymph Node* | 295 | 1.15 (0.73-1.82) | 0.55 |
| *IR Intermediate vs. good* | 295 | 1.83 (1.03-3.26) | 0.039 | *IR Intermediate vs. good* | 295 | 1.41 (0.69-2.86) | 0.35 |
| *IR Poor vs. good* | 295 | 3.05 (1.67-5.57) | 0.00029 | *IR Poor vs. good* | 295 | 2.86 (1.4-5.86) | 0.0041 |
| **Multivariate Clinical, Histological and 70-gene NKI Signature Analysis** | | | | | | | |
| *Size in mm* | 295 | 1.03 (1.01-1.06) | 0.005 | *Size in mm* | 295 | 1.03 (1.0-1.06) | 0.037 |
| *Elston Ellis Grade II vs. I* | 295 | 1.5 (0.74-3.06) | 0.26 | *Elston Ellis Grade II vs. I* | 295 | 2.51 (0.84-7.46) | 0.098 |
| *Elston Ellis Grade III vs. I* | 295 | 1.61 (0.77-3.38) | 0.2 | *Elston Ellis Grade III vs. I* | 295 | 3.04 (1.01-9.12) | 0.048 |
| *Estrogen receptor* | 295 | 1.05 (0.67-1.66) | 0.82 | *Estrogen receptor* | 295 | 0.64 (0.39-1.03) | 0.065 |
| *Lymph Node* | 295 | 1.24 (0.82-1.87) | 0.3 | *Lymph Node* | 295 | 1.13 (0.72-1.78) | 0.6 |
| *NKI Poor vs. good* | 295 | 3.84 (2.1-7.05) | 0.000013 | *NKI Poor vs. good* | 295 | 5.01 (2.15-11.67) | 0.00019 |

**Supplemental Figure Legends**

**Supplementary Figure 1a**

Kaplan–Meier analysis of BCSS. The analysis was performed for 403 patients. In all cases except the Intrinsic/UNC subtyping (HU) the blue (red) curve represents the ‘good’ (‘poor’) outcome group. Each panel depicts the result obtained after applying one of the classifiers (indicated in the heading) as described in the Methods section. The represented signatures are T17 & T52: the Molecular Prognostic Index signature (Teschendorff et al.); IGS: the Invasiveness Gene Signature (Liu et al.); Hu: the Intrinsic/UNC gene set (Hu et al.) resulting in the Luminal A (blue), Normal-like (coral), Luminal B (red) Basal (grey) and Her2 (purple) subtypes; CSR: the Core Serum Response signature (Chang et al.); CIN70 & CIN25: the Chromosomal Instability Signature (Carter et al.); Gene expression Grade Index (Sotiriou et al., GGI) and Wang: the 76-gene signature (Wang et al.).

**Supplementary Figure 1b**

Top panel: Performance of the signatures on subgroups of the patient population. The vertical axis represents the –log(p-value) of the logrank test from the Kaplan-Meier analysis for a particular subgroup with BCSS as endpoint. The analysed subgroups are: LN negative (lymph node negative); LN positive (lymph node positive); ER positive (estrogen receptor positive), ER negative (estrogen receptor negative); Low Grade (Elston Ellis I); High Grade (Elston Ellis III); individual Miller and Pawitan datasets. The horizontal line represents a p-value of 0.05.

Bottom panel: Time censoring performance analysis of the signatures. The horizontal axis represents the time at which right-censoring was applied to all samples. The vertical axis represents the –log(p-value) of the log rank test from the Kaplan-Meier analysis for a given time-censoring and a particular signature with BCSS as endpoint. The horizontal line represents a p-value of 0.05.

**Supplementary Figure 2**

Heatmaps showing the concordance of the nine classifiers across clinical subgroups among the 1127 human breast tumor samples. Clinical parameters and the classification of each signature are represented in the rows, each sample in a column. Samples with DMFS as end point are shown on the left, BCSS on the right. Further stratifications were made for ER and LN status. Black: event; White: no event; Grey: missing data; Red: positive; Green: negative; Yellow: classified as good; Blue: classified as poor. ERneg: Estrogen receptor negative; ERpos: Estrogen receptor positive; Conc: concordant; Disc: discordant.

**Supplementary Figure 3**

Overlap and performance analysis of 403 samples with BCSS as endpoint.

**Supplementary Figure 3a**

Distribution of the samples as a function of the number of times a sample was classified as ‘poor’ prognosis by the gene signatures.

**Supplementary Figure 3b**

Distribution of metastasis events as a function of the number of times a sample was classified as ‘poor’ prognosis by the gene signatures.

**Supplementary Figure 3c**

Distribution of ER negative samples as a function of the number of times a sample was classified as ‘poor’ prognosis by the gene signatures.

**Supplementary Figure 3d**

Distribution of HER2 amplified samples as a function of the number of times a sample was classified as ‘poor’ prognosis by the gene signatures.

**Supplementary Figure 3e**

Tumour size (Mean, 5th, 25th, 75th and 95th percentile) as a function of the number of times a sample was classified as ‘poor’ prognosis by the gene signatures.

**Supplementary Figure 3f**

Distribution of Grade I, II, III samples (Elston Ellis) as a function of the number of times a sample was classified as ‘poor’ prognosis by the gene signatures. Dark: Grade I. Grey: Grade II, White: Grade III.

**Supplementary Figure 3g**

Average predictive accuracy (Percentage of samples well classified) as a function of the number of times a sample was classified as ‘poor’ prognosis by the gene signatures.

**Supplementary Figure 4**

Each cell in this matrix represents the –log10(p-value) of the logrank test on the Chin-Loi training set for each pair-wise combination of the module classifiers. The dichotomous outputs (‘poor’ or ‘good’ outcome) of the individual classifiers were combined in three outcome classes: low, intermediate and high risk, depending on whether the individual classifiers had a concordant ‘good’ outcome, were discordant or a concordant ‘poor’ outcome, respectively.

**Supplementary Figure 5**

Left most column: Kaplan-Meier plots on the van de Vijver data for the subgroups defined by the NPI (first row), StGallen (second row), and Adjuvant! (third row) clinical staging systems. Remaining columns: Kaplan-Meier plots for the Immune-RNA module classifier within each of the clinical subgroups for each staging system.
