# Supplementary figures and images for: A comprehensive analysis of prognostic signatures reveals the high predictive capacity of the Proliferation, Immune response and RNA splicing modules in breast cancer
Source: Breast Cancer Res. 2008 Nov 13;10(6):R93. doi: 10.1186/bcr2192 (PMC2656909; doi:10.1186/bcr2192)

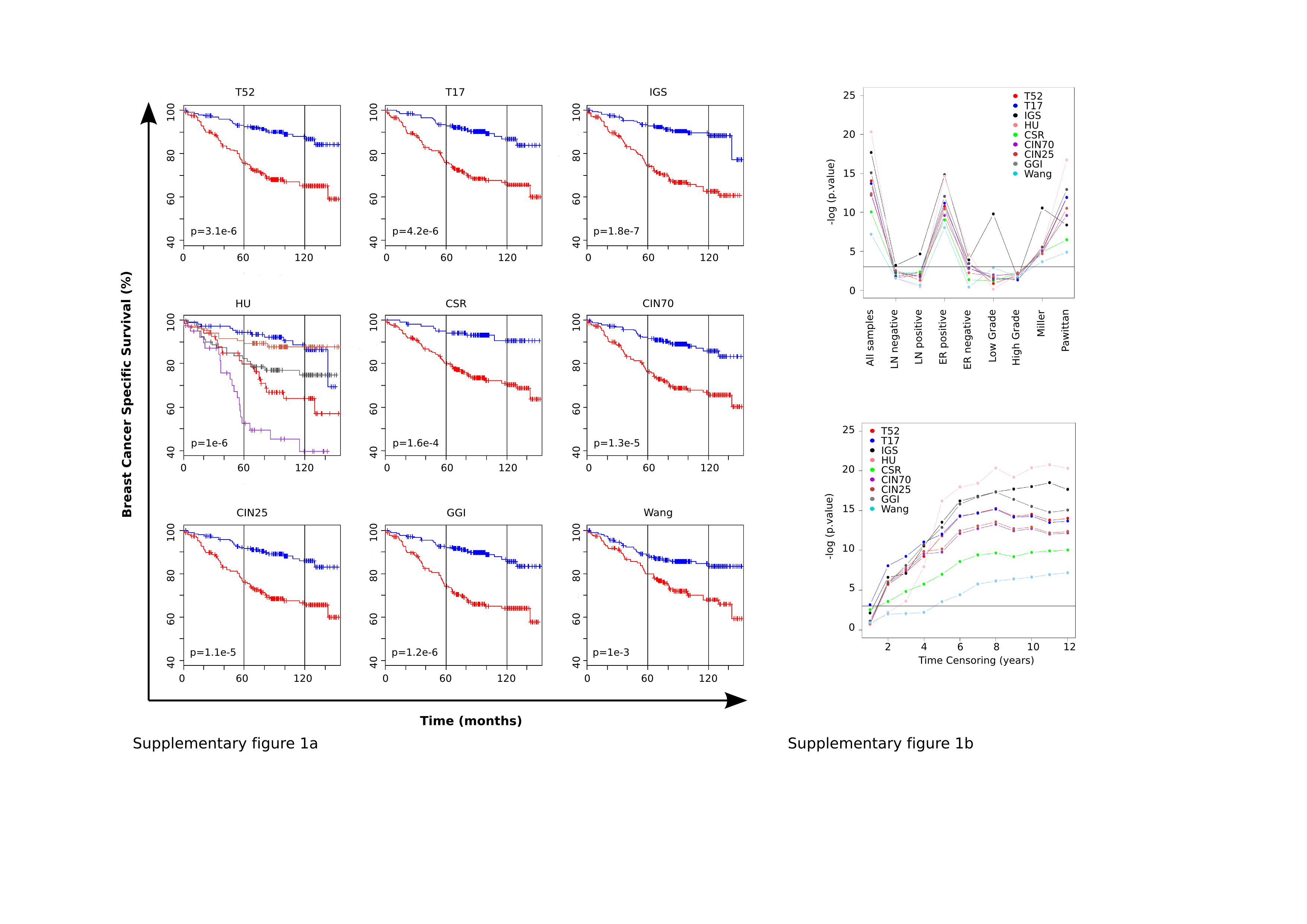

Supplement: Additional file 2 — An Adobe file containing a figure of the RNA splicing, Immune and 72 Proliferation gene annotations [Probe_ID, EntrezID, OMIM, Ensembl, UnigeneID, Representative Public ID, RefSeq Transcript ID, Gene Symbol, k-means metastasis, k-means no metastasis]. [file bcr2192-S2.png]

# Supplementary Figure 2

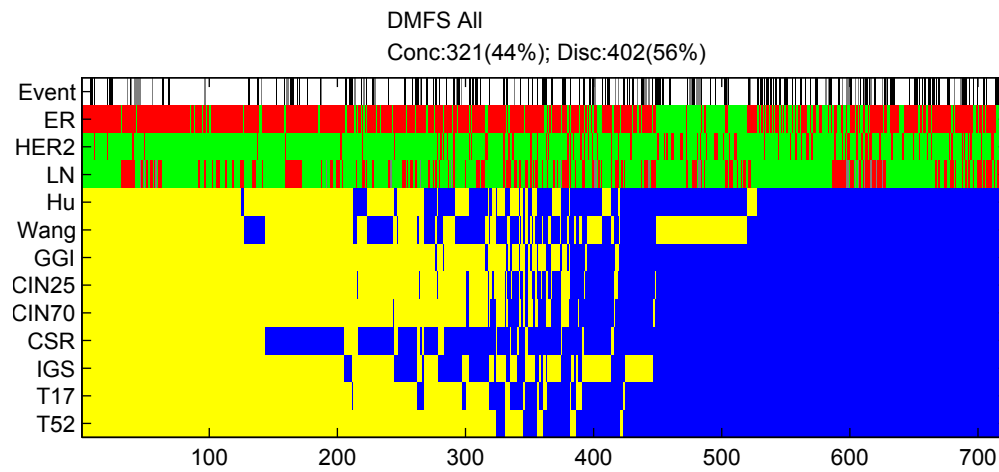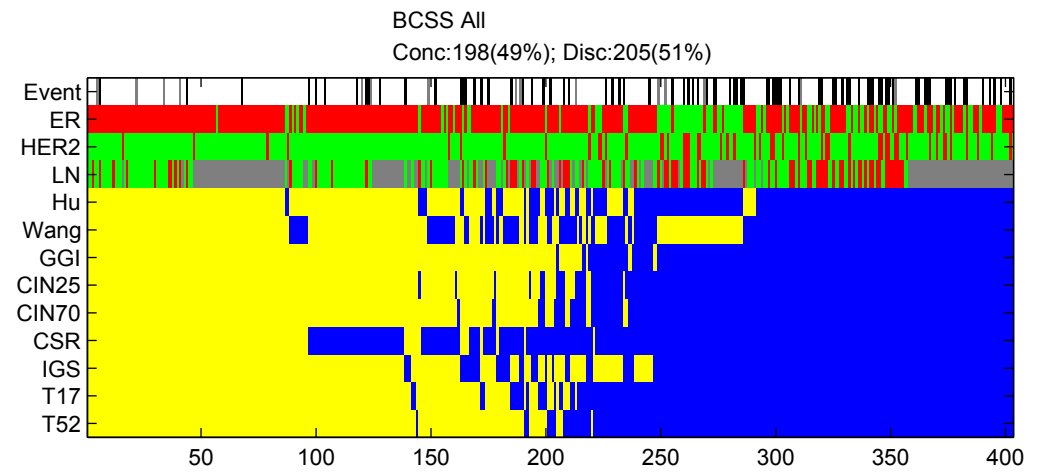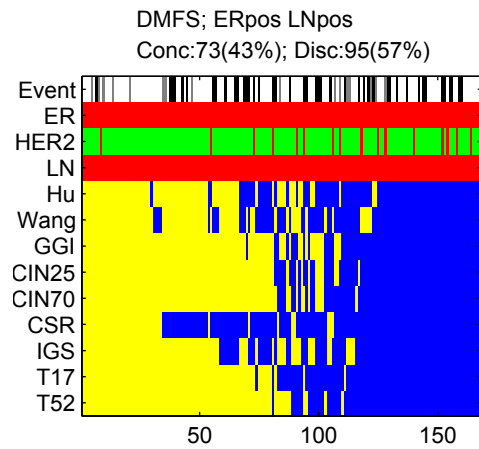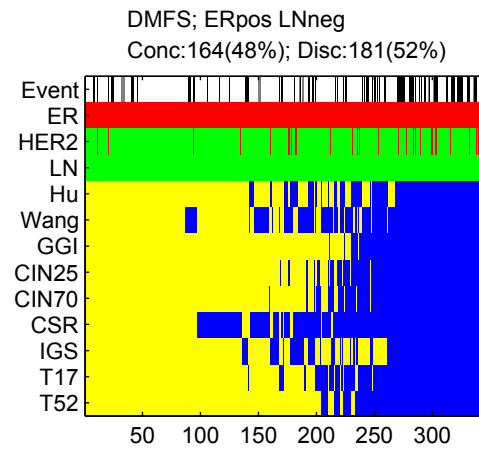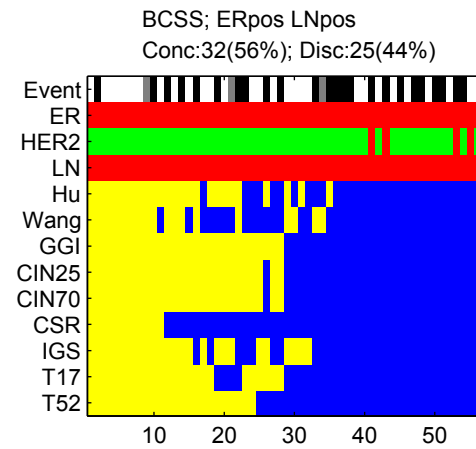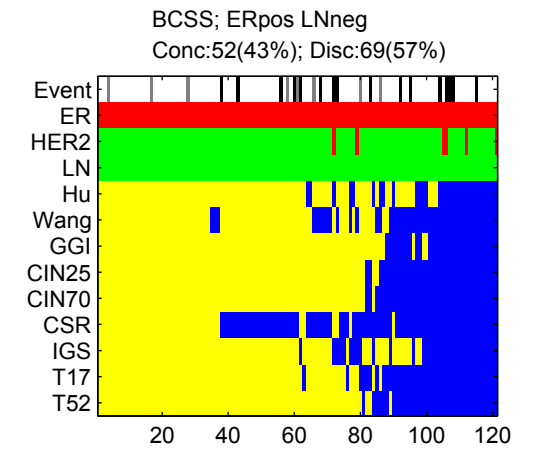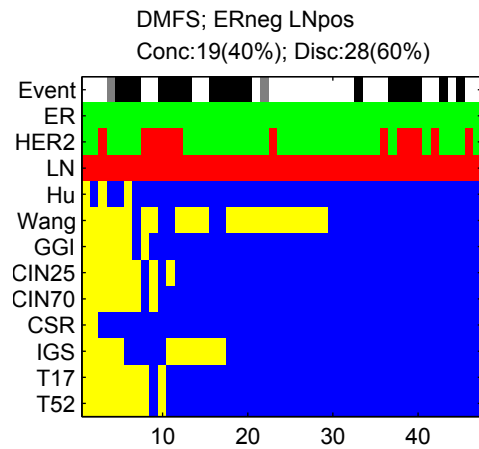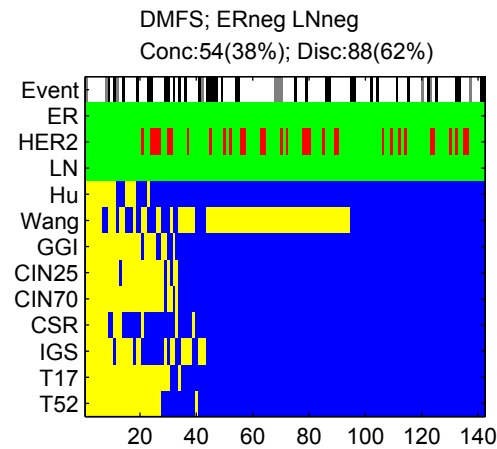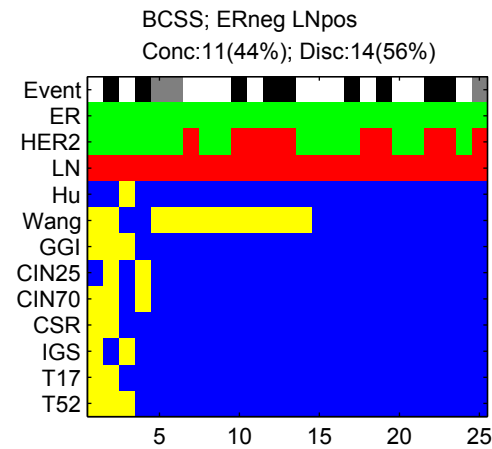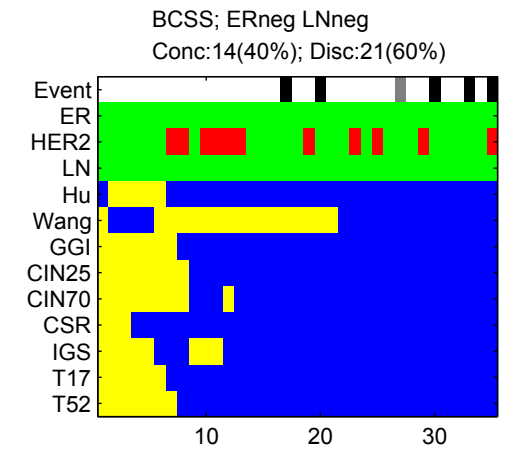

Supplement: Additional file 3 — An Adobe file containing a figure showing the nine signature Kaplan–Meier curves with BCSS as endpoints (S1a), showing the performance of the signatures on subgroups of the patient population (S1b (top panel)), and showing) the time-censoring performance analysis of the signatures (1b (bottom panel). [file bcr2192-S3.pdf]

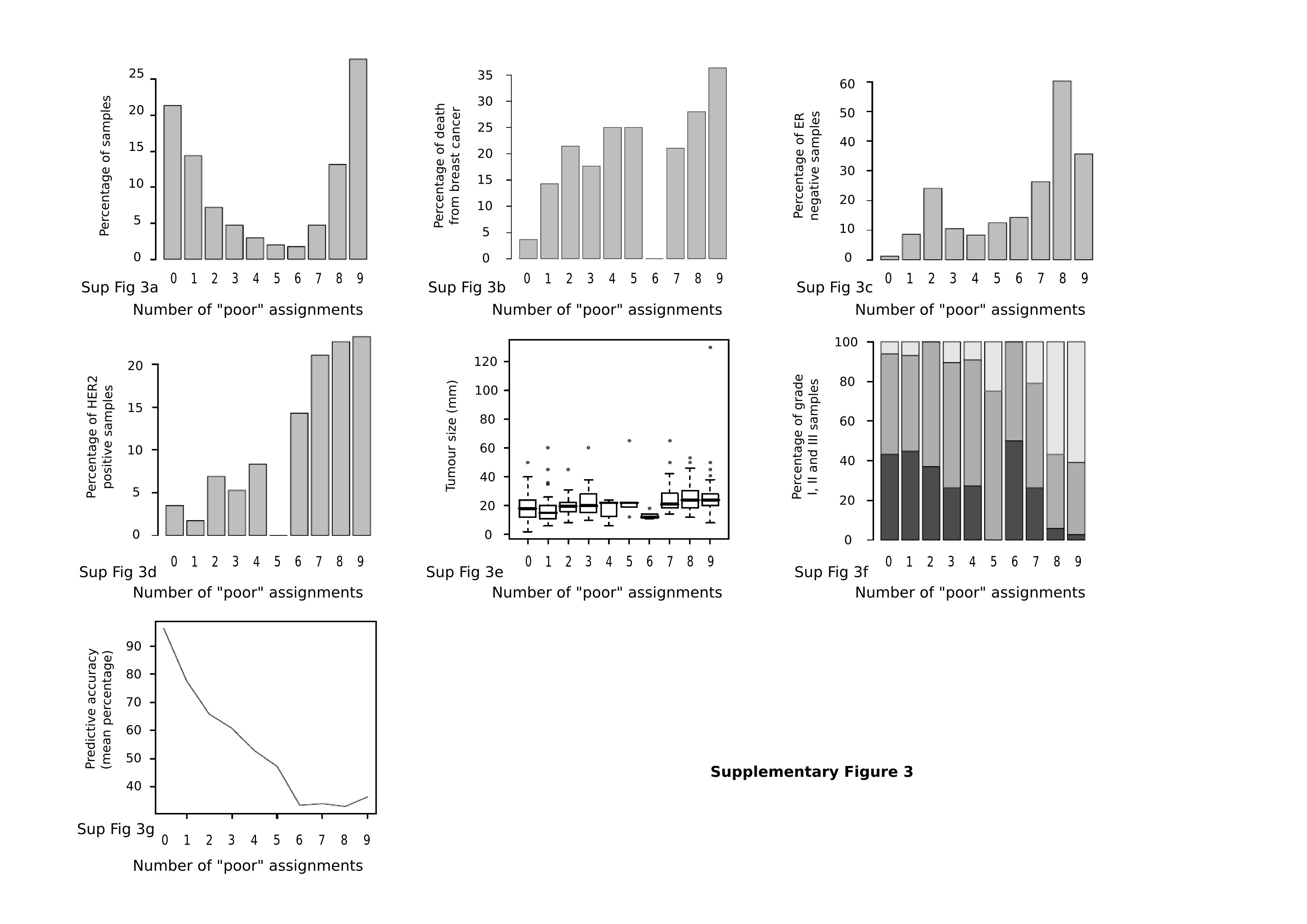

Supplement: Additional file 4 — An image file containing a figure showing heatmaps of the concordance of the nine classifiers across clinical subgroups among the 1,127 human breast tumor samples. [file bcr2192-S4.png]

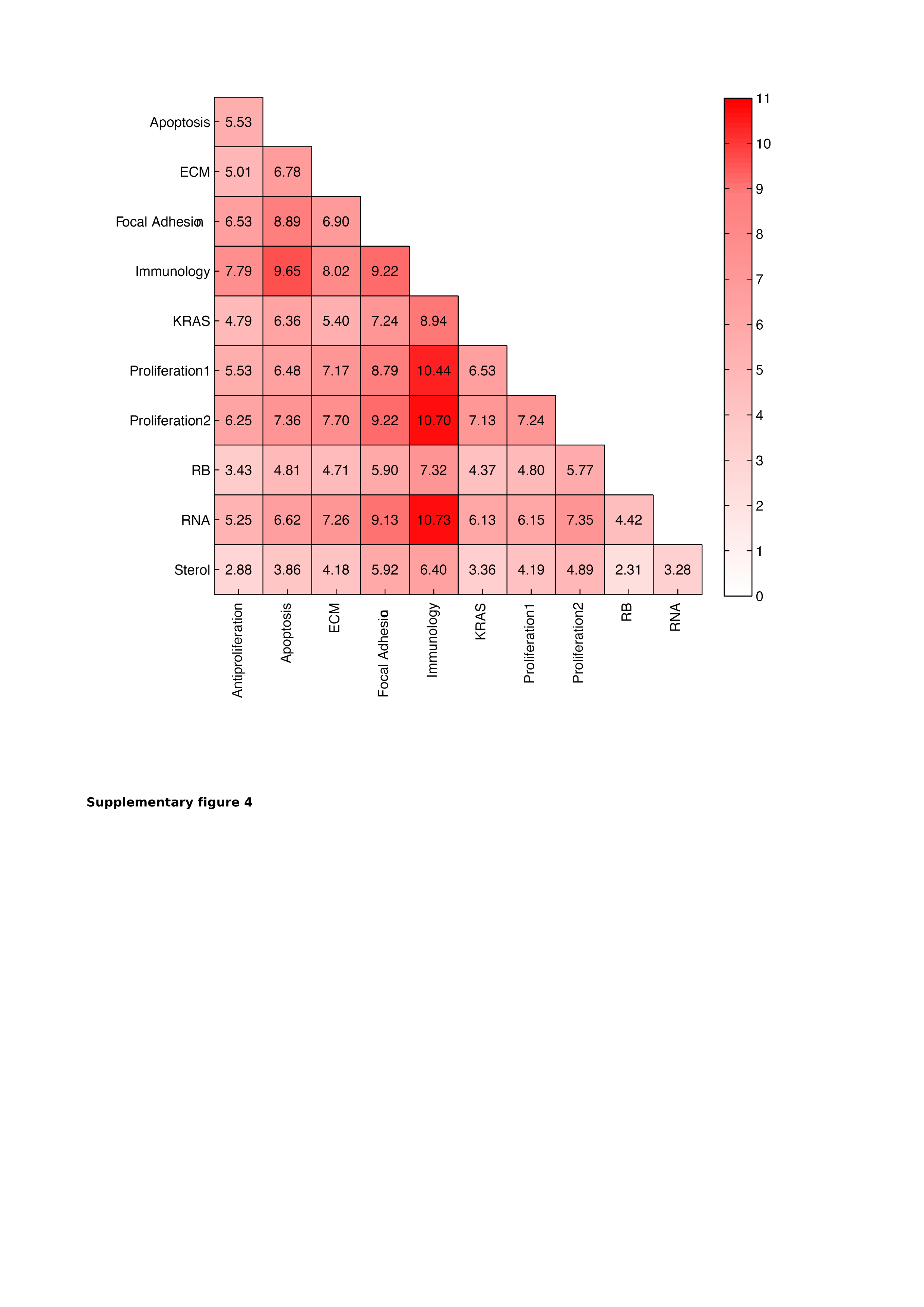

Supplement: Additional file 5 — An image file containing a figure showing the overlap and performance analysis of 403 samples with BCSS as the endpoint. [file bcr2192-S5.png]
