# Supplementary material for: A comprehensive analysis of prognostic signatures reveals the high predictive capacity of the Proliferation, Immune response and RNA splicing modules in breast cancer
Source: Breast Cancer Res. 2008 Nov 13;10(6):R93. doi: 10.1186/bcr2192 (PMC2656909; doi:10.1186/bcr2192)
Supplement: Additional file 7 — An Adobe file containing a figure showing the Kaplan–Meier plots on the dataset of van de Vijver and colleagues for the subgroups defined by the Nottingham Prognostic Index, St Gallen, and AdjuvantOnline! clinical staging systems – plots for the Immune/RNA splicing module classifier within each of the clinical subgroups for each staging system. [file bcr2192-S7.pdf]

KM curves of the RNA−Immune Module classifier within NPI/StGallen/Adjuvant subgroups of the Vijver dataset

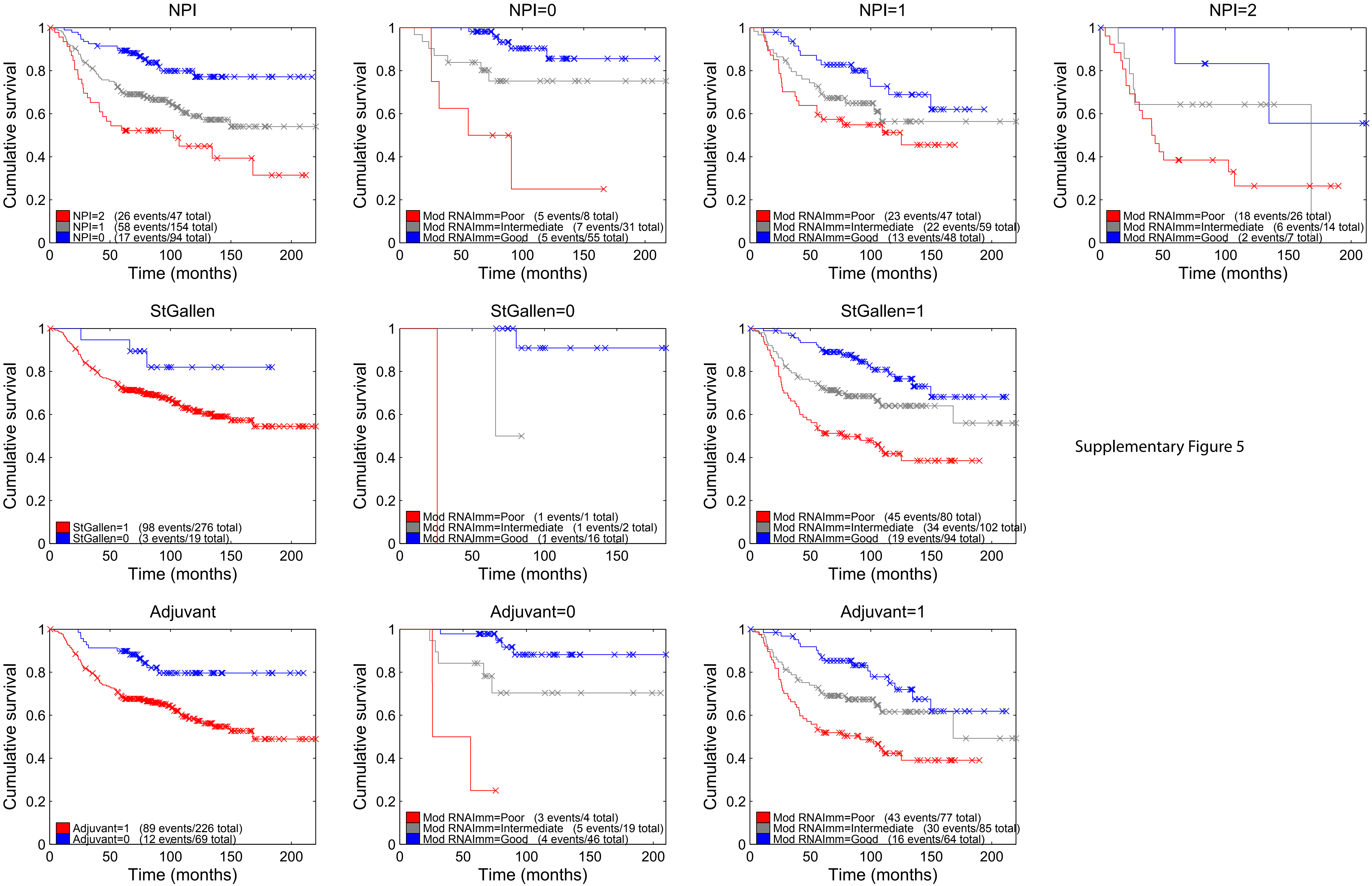

Supplementary Figure 5
